# A Comprehensive Census of Microbial Diversity in Hot Springs of Tengchong, Yunnan Province China Using 16S rRNA Gene Pyrosequencing

**DOI:** 10.1371/journal.pone.0053350

**Published:** 2013-01-09

**Authors:** Weiguo Hou, Shang Wang, Hailiang Dong, Hongchen Jiang, Brandon R. Briggs, Joseph P. Peacock, Qiuyuan Huang, Liuqin Huang, Geng Wu, Xiaoyang Zhi, Wenjun Li, Jeremy A. Dodsworth, Brian P. Hedlund, Chuanlun Zhang, Hilairy E. Hartnett, Paul Dijkstra, Bruce A. Hungate

**Affiliations:** 1 State Key Laboratory of Biogeology and Environmental Geology and Institute of Earth Sciences, China University of Geosciences, Beijing, China; 2 Department of Geology and Environmental Earth Science, Miami University, Oxford, Ohio, United States of America; 3 School of Life Sciences, University of Nevada, Las Vegas, Nevada, United States of America; 4 State Key Laboratory of Biogeology and Environmental Geology, China University of Geosciences, Wuhan, Hubei Province, China; 5 Key Laboratory of Microbial Diversity in Southwest China of Ministry of Education and Laboratory for Conservation and Utilization of Bio-resources of Yunnan Institute of Microbiology, Yunnan University, Kunming, Yunnan Province, China; 6 State Key Laboratory of Marine Geology, School of Ocean of Earth Sciences, Tongji University, Shanghai, China; 7 Department of Marine Sciences, the University of Georgia, Athens, Georgia, United States of America; 8 School of Earth and Space Exploration and Department of Chemistry and Biochemistry, Arizona State University, Tempe, Arizona, United States of America; 9 Department of Biological Sciences, Northern Arizona University, Flagstaff, Arizona, United States of America; J. Craig Venter Institute, United States of America

## Abstract

The Rehai and Ruidian geothermal fields, located in Tengchong County, Yunnan Province, China, host a variety of geochemically distinct hot springs. In this study, we report a comprehensive, cultivation-independent census of microbial communities in 37 samples collected from these geothermal fields, encompassing sites ranging in temperature from 55.1 to 93.6°C, in pH from 2.5 to 9.4, and in mineralogy from silicates in Rehai to carbonates in Ruidian. Richness was low in all samples, with 21–123 species-level OTUs detected. The bacterial phylum *Aquificae* or archaeal phylum *Crenarchaeota* were dominant in Rehai samples, yet the dominant taxa within those phyla depended on temperature, pH, and geochemistry. Rehai springs with low pH (2.5–2.6), high temperature (85.1–89.1°C), and high sulfur contents favored the crenarchaeal order *Sulfolobales,* whereas those with low pH (2.6–4.8) and cooler temperature (55.1–64.5°C) favored the *Aquificae* genus *Hydrogenobaculum*. Rehai springs with neutral-alkaline pH (7.2–9.4) and high temperature (>80°C) with high concentrations of silica and salt ions (Na, K, and Cl) favored the *Aquificae* genus *Hydrogenobacter* and crenarchaeal orders *Desulfurococcales* and *Thermoproteales*. *Desulfurococcales* and *Thermoproteales* became predominant in springs with pH much higher than the optimum and even the maximum pH known for these orders. Ruidian water samples harbored a single *Aquificae* genus *Hydrogenobacter*, whereas microbial communities in Ruidian sediment samples were more diverse at the phylum level and distinctly different from those in Rehai and Ruidian water samples, with a higher abundance of uncultivated lineages, close relatives of the ammonia-oxidizing archaeon “*Candidatus* Nitrosocaldus yellowstonii”, and candidate division O1aA90 and OP1. These differences between Ruidian sediments and Rehai samples were likely caused by temperature, pH, and sediment mineralogy. The results of this study significantly expand the current understanding of the microbiology in Tengchong hot springs and provide a basis for comparison with other geothermal systems around the world.

## Introduction

Tengchong County, located in Yunnan Province, Southwestern China, is known for its geothermal features [1]–[3]. Tectonically, Tengchong is located at the collision boundary between the India and Eurasia plates. Subduction of oceanic crust leads to extensive volcanism. The hot springs of Tengchong are a result of such volcanism with more than 50 volcanoes and 140 geothermal areas throughout the county [4]. In terms of the diversity and scale, the Tengchong springs are comparable to the geothermal systems in Yellowstone National Park (YNP) [5], Japan [6], and Kamchatka, Russia [7].

The Rehai (“Hot Sea”) and Ruidian geothermal fields in Tengchong are two regions of intense hydrothermal activity with numerous springs and pools. Physicochemical conditions span a wide range of temperature (58 to ∼97°C) and pH (<1.8 to ≥9.3) and these conditions provide numerous niches to support phylogenetically and functionally distinct microbial communities. Therefore, the Tengchong geothermal fields likely represent a biodiversity hotspot for thermophiles and provide an opportunity to compare microbial diversity and community structure with other geothermal areas around the world. Past studies in this area have focused on cultivation, physiology, and biotechnological applications of thermophilic microorganisms (reviewed in [8]). Overall, these studies revealed that acidic springs harbor thermoacidophilic bacteria and archaea, particularly *Sulfolobales*, which can grow chemolithotrophically by oxidizing sulfide, sulfur, thiosulfate and ferrous iron. Likewise, alkaline springs harbor many alkaliphilic bacteria, particularly *Firmicutes* and *Thermales*, which grow chemoorganotrophically on a number of organic substrates.

Whereas cultivation-dependent studies are valuable for isolating novel organisms and exploring their properties, the cultivation-independent methods offer a more comprehensive assessment of microbial diversity [9]. In Tengchong, however, only a few molecular-based studies have been reported. Specifically, three orders of *Crenarchaeota* (*Sulfolobales, Desulfurococcales*, and *Thermoproteales*) were found in Tengchong springs and crenarchaeotal diversity was correlated with temperature [10]. Microbial diversity in two hot spring microbial mats from Tengchong was recently described [11]. Other cultivation-independent studies have focused on ammonia-oxidizing archaea (AOA) [12]–[14] and these studies suggest that AOA may be a minor but potentially active component of the microbial community in several Tengchong springs.

Despite these previous efforts, a comprehensive census of the microbial communities in Tengchong hot springs is still lacking. The primary objective of this study was to census a large number of high temperature springs in Tengchong and to investigate the relationships between thermophilic microbial communities and physicochemical conditions. To achieve this objective, a coordinated geochemical and molecular survey was conducted for 16 Tengchong hot springs and the relationships among microbial diversity, community structure, and geochemistry were explored. The results of this study expand the current understanding of the microbiology in Tengchong hot springs and provide a basis for comparison with other geothermal systems around the world.

## Experimental Procedures

### Field Measurements and Spring Selection

No specific permits were required for the described field studies because no animal or human subjects were involved in this research. The sampling locations are not privately owned or protected in any way. The field studies did not involve endangered or protected species.

**Figure 1:**
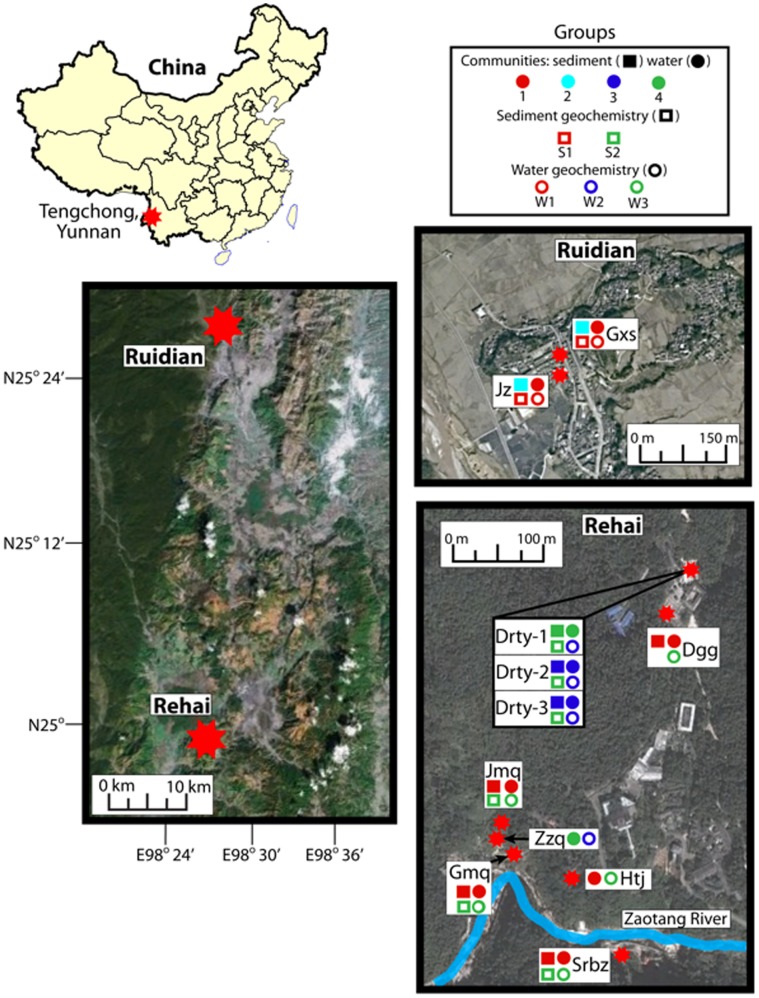
A map that shows the nine springs from both the Ruidian and Rehai locations. The open symbols represent the geochemistry and the closed symbols represent the microbial communities. The circles represent the water and the squares represent the sediment geochemistry. The colors of each symbol represent the grouping of either microbial communities (from Figure 2) or water and sediment geochemistry (from Figure S1 and S3 for water and sediment/sinter, respectively) based on hierarchical clustering. The microbial community, sediment geochemistry, and water geochemistry were analyzed separately and color comparisons across different symbols cannot be made; however, a similar color within one symbol (for example solid circle) depicts microbial community or geochemical similarities across the sampling sites. XRD mineralogical analysis of the sediment geochemistry at Dagunguo (Dgg) failed. Huitaijing (Htj) did not have any sediment. There were no microbial data for the Zhenzhuquan (Zzq) sediments because of PCR failure. Abbreviations are Jimeiquan (Jmq), Zhenzhuquan (Zzq), Guminquan (Gmq), Huitaijing (Htj), Jinze (Jz), Diretiyanqu (Drty), Gongxiaoshe (Gxs), Shuirebaozhaqu (Srbz), and Dagunguo (Dgg).

Field measurements and sample collections were conducted in January 2011. Water temperature, pH, conductivity (portable meters; LaMotte, MD, USA) and the spring size were measured for more than 20 springs. Sixteen springs were chosen for further study, including 14 from Rehai and 2 from Ruidian (Figure 1 and Figure S1). Concentrations of ammonium (NH_4_^+^), total sulfide (ΣS^2−^), ferrous iron (Fe^2+^), nitrate (NO_3_^−^) and nitrite (NO_2_^−^) were measured with spectrophotometry using Hach test kits (Hach Chemical Co., IA, USA) after filtering spring water through 0.20 µm polyethersulfone (PES) membrane filters (Pall Corp., NY).

### Sample Collection

Sixteen water samples, 1 microbial streamer, 13 sediment samples, and 6 sinter samples from 16 springs were collected for geochemical and microbiological analyses (Table 1). Depending on the site, 50–360 mL of spring waters were filtered through a syringe filter (0.22 µm pore size, 25 mm diameter, Millipore, USA) to collect biomass. In one spring (Dagunguo), biomass was also collected via tangential flow filtration (TFF) that concentrated 200 L of water to 2 L using a 30 kDa MWCO prep/scale TFF-6 filter (Millipore, USA) followed by centrifugation at 12000×g (4°C, 20 min.) after four days of storage in the lab at 4°C.

**Table 1:** Sample codes, spring name, type and size; sample type; and selected geochemical parameters of spring water.

Samplecode	Pingyin name – English name	pH	Temp°C	GPS location	Descriptions
Dgg	Dagunguo - Great Boiling Pot	7.20	84.5	N24.95344° E98.43780°	The largest and most prominent spring at Rehai. Roughly cylindrical with diameter 555–590 cm and depth ∼150 cm, with two vigorous degassing sources. Sinter composed of amorphous silicate. Sample type includes sinter, water.
Drty-1	Diretiyanqu - Experimental Site (in fault, just under cliff)	2.58	85.1	N24.95396° E98.43829°	Small acid sulfate pool with small outlet. Irregularly shaped with length ∼ 63 cm, width ∼ 56 cm, and depth ∼ 6.5 cm, with vigorously degassing sources. Turbid. Mixed with fine silicate sands at bottom. Sample type includes sediment and water.
Drty-2	Diretiyanqu - Experimental Site (below Drty-1)	2.57	64.5	N24.95363° E98.43891°	Small acid sulfate pool with slow outflow. Roughly round-shaped with diameter 40–50 cm, and depth 10.5 cm. Mixed with fine silicate sands at bottom. Sample type includes sediment and water
Drty-3	Diretiyanqu - Experimental Site (On the right side from Drty-1)	2.46	55.1	N24.95389° E98.43834°	Small acid sulfate pool with slow outflow. Irregularly shaped with length ∼ 51 cm, width ∼ 26 cm, and depth ∼ 5.5 cm, with some degassing. Grey-white water from suspended clays. Also silicate sands at bottom. Sample type includes sediment and water.
GmqS	Gumingquan– Drum Beating Spring (source)	9.35	93.0	N24.57060°E98.43615°	Small source pool with a high flow rate (10.4 L/S); with length ∼ 98 cm, width ∼ 79 cm, and depth ∼9.5 cm. Clear water. Some silicate sands at bottom. Sample type includes sediment and water.
GmqC	Gumingquan – Drum Beating Spring (right channel)	9.36	89.0	N24.57060°E98.43615°	Shallow water flow channel (∼ 20 cm width, ∼ 5 cm depth) downstream of GmqS. Clear water and rocky bottom. Sample type includes sediment and water.
GmqP	Gumingquan – Drum Beating Spring (streamer pool)	9.30	82.5	Close to GmqC	Pool downstream of GmqS, with a depth ∼ 16 cm. Many pink streamers. Clear water. Mixed with fine silicate sands at bottom. Sample type includes sediment, water and streamer.
JmqL	Jiemeiquan - Sisters Spring (Left)	9.25	93.6	N24.95112°E98.43600°	A shallow pool with spouting spring source and outflow. Length and width 100–110 cm, and depth ∼9.5 cm. Lots of red or green mats on the bank of the spring. Clays and sands at the bottom. Sample type includes sediment and water.
JmqR	Jiemeiquan - Sisters Spring (Right)	9.39	83.2	Close to JmqL	A shallow pool with a small source, downstream of JmqL. Length ∼950 cm, width ∼100 cm, and depth 4.5 cm. Lots of pink streamers. Sands and clays at bottom. Sample type includes sediment and water.
Zzq	Zhenzhuquan - Pearl Spring	4.79	89.1	N24.95115°E98.43596°	Constructed into a shallow, heart-shaped pool with the longest dimension of 436 cm, and a depth ∼6–7 cm. Vigorously degassing. No visible outflow. Silicate sands at bottom. Sample type includes sediment and water.
HtjL	Huitaijing - Pregnancy Spring (Left)	8.11	90.0	N24.95089°E98.43664°	Constructed into cylindrical well with diameter 65 cm, and depth 63 cm. Visible particles in water. Flow path is sculpted; slow outflow. Sample type includes water.
HtjR	Huitaijing - Pregnancy Spring (Right)	8.05	92.3	Near HtjL	Similar to HtjL. Sample type includes water.
SrbzU	Shuirebaozha - Hydrothermal Explosion (upstream)	8.04	79.8	N24.95002°E98.43728°	Irregularly shaped pool with many bubbling sources. The pool was also fed by rain water. The size was dependent on weather in a given season. Length ∼ 300 cm, width ∼ 240 c. Several hydrothermal explosions occurred in the past. Grey mud at the bottom. Sample type includes sediment and water.
SrbzD	Shuirebaozha - Hydrothermal Explosion (downstream)	8.28	78.2	Near SrbzU	Located near the middle of the west pool. Sample type includes sediment and water.
GxsS	Gongxiaoshe – Co-op Hotel (side)	7.29	73.8	N25.44012°E98.44081°	A large spring in the Ruidian Geothermal area. Constructed into an octagonal pool, with diameter ∼ 297 cm and depth ∼130 cm. Sinters growing at the inner wall. Soft cream-colored sediment containing carbonates at bottom. Vigorous degassing sources. No outflow, but the water was pumped out for showering by local residents. Carbonate-dominated sediments from the bottom of the pool were collected. Sample type includes sediment and water.
GxsB	Gongxiaoshe – Co-op Hotel (bottom)	7.29	73.8	Near GxsS	Sinter samples chipped from the sides of the spring. Sample type includes sinter and water.
Jz	Jinze - Golden Pond Motel	6.71	81.6	N23.44138°E98.46004°	Constructed into a cubic well with side lengths ∼ 127 and ∼134 cm and depth ∼ 103 cm, covered with a shed. Outflow was stored in a cubic tank for showering. Black mud at bottom. Sample type includes sediment and water.

Water samples were collected for the measurements of hydrogen and oxygen isotopic composition and concentrations of cation, anion, trace elements, dissolved organic carbon (DOC), and total nitrogen (TN). Water samples for hydrogen and oxygen isotope measurements were filtered through 0.22 µm polyethersulfone (PES) filters (25 mm diameter; Pall Corp., USA) and collected into 25-mL glass bottles. Water samples were collected to determine concentrations of major cations, anions, and trace elements by filtering 25 mL of water through 0.22 µm PES filters into 60-mL high density polyethylene (HDPE), acid-washed Nalgene® bottles. High-purity nitric acid (2%, V/V) was added to stabilize the trace element samples. Water samples for dissolved organic carbon (DOC) and total nitrogen (TN) measurements were filtered through the same 0.22 µm PES filters. The DOC/TN samples were stored in acid-washed and rinsed 125 mL fluorinated high-density polyethylene bottles that were pre-acidified with 0.2% (V/V) HCl.

Sediment samples were collected with sterile spatulas and spoons, and homogenized in a pre-sterilized aluminum pan. In springs with little dispersed sediment, fragments of sinter were collected using a sterilized hammer and chisel. Multiple aliquots of sediment or crushed sinter material were placed into 1.5 mL or 50 mL polypropylene tubes. All samples for microbial analysis were immediately frozen in liquid nitrogen or dry ice, stored on dry ice during transportation and at −80°C in the laboratory. Sediment samples for geochemical analyses were stored on ice until analysis.

### Geochemical Analyses

Anion concentrations were determined via ion chromatography with a Dionex DX-500 chromatograph (AS14A column, with 10 µM Na_2_CO_3_/NaHCO_3_ as an eluent, Dionex, USA). Cation concentrations were determined by direct current plasma emission spectrometry (DCP-OES, Beckman, USA). Trace element concentrations were determined by inductively coupled plasma – mass spectrometry (ICP-MS). DOC and TN concentrations were analyzed by high-temperature combustion [15], [16] with NDIR and chemiluminescence detection for C and N, respectively (TOC-V, Shimadzu Corp., Japan). Oxygen and hydrogen isotopic compositions were measured using isotope ratio mass spectrometry after CO_2_-H_2_O equilibration for oxygen and reduction to H_2_ gas for hydrogen, respectively [17].

Frozen sediments were thawed in the lab and dried at 105°C overnight. Total nitrogen (TN) and total organic carbon (TOC) was determined using a NC 2100 Elemental Analyzer interfaced with a Finnigan Delta Plus XL isotope ratio mass spectrometer. TOC was determined by coupled elemental analysis followed by isotope ratio mass spectrometry after fumigating sediment with HCl to remove carbonates. X-ray diffraction was performed to identify the mineralogy using a Scintag X1 powder diffractometer system (Bruker Corporation, USA) according to the prodecure described elsewhere [18].

### DNA Extraction

DNA was extracted from biomass-containing filters or from 0.5 g of sediment using FastDNA SPIN Kit for Soil (MP Biomedical, OH, USA) with a final elution in 70 µL de-ionized water. DNA concentration and quality were assessed based on spectral absorbance at 260 nm wavelength and absorbance ratios of 260/280 nm and 260/230 nm, respectively, using a NanoDrop ND-1000 Spectrophotometer (NanoDrop Technology, USA). DNA samples were divided into 20 µL aliquots and preserved at −80°C until further processing.

### Amplification of 16S rRNA Genes

The bacterial and archaeal V4– V8 variable regions of the 16S rRNA gene were amplified with the modified forward primer 515F (5′-GTGYCAGCMGCCGCGGTAA-3′) in combination with the reverse primer 1391R (5′-GACGGGCGGTGWGTRCA-3′) [19]–[21]. The modified 515F primer was based on the published forward primer 515F (5′-GTGCCAGCMGCCGCGGTAA-3′) and the consideration that the change from C to Y in the 4^th^ position increased the number of matches from <1% to >83% of crenarchaeotal sequences in the Ribosomal Database Project [22]. To assess the effect of this change, seven samples (Dagunguo, Diretiyanqu-3, Gumingquan, Jiemeiquan, Shuirebaozha, Gongxiaoshe water and sinter, Table 1) were amplified with both the original and the modified 515F primers.

In order to identify the samples from the reads, unique 8-bp barcodes were added at the 5′-end of both the forward and reverse primers. The polymerase chain reaction (PCR) mix contained 10 ng of template DNA, 5 µl rTaq reaction buffer, 400 nM of each primer, 200 µM dNTPs, and 0.3 unit of rTaq polymerase (Takara, Dalian, China) in a 25 µl reaction. The amplification procedure was as follows: an initial denaturation step at 95°C for 5 min, and 30 cycles of denaturing at 94°C for 30 s, annealing at 54°C for 30 s, and extension at 72°C for 1 min, followed by a final extension step at 72°C for 10 min. Amplicons from four PCR were pooled for each sample. PCR products were purified with the Qiagen gel extraction kit (Qiagen, USA) and quantified on a QuBit 2.0 fluorometer (Invitrogen Corp., USA). Finally, amplicons of all samples were pooled in equimolar concentrations for pyrosequencing.

### Pyrosequencing and Data Analysis

Pyrosequencing of the V4 region of the 16S rRNA gene was carried out from the 515F-end of the amplicons with a GS FLX sequencer (454 Life Sciences, USA) at the Chinese National Human Genome Center in Shanghai. Quality screening was completed in Mothur by removing low quality reads [23]. All the remaining reads were de-noised [24] and trimmed to a uniform length of 238 bp, followed by clustering analysis at the similarity levels of 80%, 90%, 95% and 97% using UCLUST [25] in the QIIME (quantitative insights into microbial ecology) pipeline [26]. The most abundant sequence from each cluster was chosen as a representative. All representative sequences were aligned with the PyNAST method [27], and chimeric operational taxonomic units (OTUs) were identified with Chimera Slayer [28] and excluded. Taxonomy was assigned to each representative sequence using BLAST with gg_97_otus_4feb2011.fasta (http://greengenes.lbl.gov; GreenGenes training set) as the reference database. All sequences in the Thaumarchaeota phylum were manually moved out of Crenarchaeota [29]. All 454 sequences have been submitted to the Short Read Archive database at NCBI (accession no. SRA056421).

### Statistical Analysis of Geochemical and Microbial Data

To identify geochemical parameters that contributed most to microbial variability, seventy-nine water geochemical variables (Table S1 and S2 minus the mineralogical data) were first subjected to principal component analysis (PCA) with the ‘vegan’ package in the R programming environment [30]. Co-correlated geochemical variables were removed, and the remaining 17 variables were rank-ordered based on their correlations to the first three axes. For the sediment geochemistry, all 18 mineralogical variables (Table S1) were used. Seventeen water geochemical variables were subjected to hierarchical clustering of Log_10_ geochemical values with linkage by the unweighted pair-group method with arithmetic mean (UPGMA) of Euclidean distances to identify groups of sites with similar water geochemistry. Eighteen sediment geochemical variables were subjected to similar clustering analysis but without logarithmic transformation of the values because all values were between 0 and 1.

Chao1 (predicted number of OTUs), Shannon, and equitability indices, based on 16S rRNA gene sequence data, were calculated at the 97% cutoff level after removing four samples with fewer than 978 reads and normalizing all remaining libraries to the one with the smallest number of reads (978 reads). These diversity indices were tested for their correlation with the geochemical data using Mantel test.

Analysis of similarity (ANOSIM) was performed, based on the Bray-Curtis dissimilarity at the 97% similarity level, to test for any significant dissimilarity or similarity in community composition among certain groups of springs defined *a priori*. The groups were pre-defined based on water and sediment geochemistry. The test was performed in three steps: First, only one factor, e.g., geothermal field (Rehai vs. Ruidian) and sample type (water vs. sediment) was considered individually. Second, geothermal field and sample type were considered together, forming four groups: Rehai water, Rehai sediment, Ruidian water, and Ruidian sediment. Third, because of the large pH range (2.5–9.4) in the Rehai hot springs, the pH factor (low: 2.5–4.8 and high: 6.7–9.4) was added, forming six groups, including Rehai low-pH water, Rehai low-pH sediment, Rehai high-pH water, Rehai high-pH sediment, Ruidian high-pH water, and Ruidian high-pH sediment. There was no low-pH water or sediment in Ruidian. Pairwise comparisons were carried out in ANOSIM and pairwise p- and r values were obtained as a way to assess any significant difference in community composition between any two of the above groups.

Jackknifed UPGMA clustering was performed to compare microbial community similarity among the samples based on the Bray-Curtis dissimilarity at the 97% similarity OTU level. For certain representative groups that were identified by ANOSIM and the UPGMA clustering, SIMPER (similarity percentage) analysis was performed to rank the top ten OTUs that contributed to the observed similarity (or dissimilarity) among two groups. The average abundances of these OTUs in each group were then calculated.

The BIO-ENV procedure [31] was used to identify the best subset of environmental variables to reveal correlations between community data (Bray?Curtis dissimilarity) and scaled environmental data (Euclidean distance) using identified 17 water and 18 sediment geochemical variables. Mantel tests were further performed to confirm correlation between the community structure and selected geochemical variables.

To identify differences between the two primer sets, ANOSIM, non-parametric multivariate ANOVA (Adonis), and multi-response permutation procedure (MRPP) were performed based on the Bray-Curtis dissimilarity distance matrices. Other than geochemical clustering analyses, all statistical analyses were done in QIIME pipeline and/or using the package “vegan” [30] in the R programming environment.

## Results

### Physical and Chemical Characteristics of Tengchong Hot Springs

Tengchong hot springs exhibited a wide range of physical and chemical conditions (Table 1 and Figure S1, Table S1 & S2) with great variations in dimension and sediment and sinter mineralogy. Visually, there were three major types of springs: 1) large pools with standing water (expected long residence time); 2) high discharge, fast flowing springs with small source pools (expected short residence time); and 3) small, shallow acidic pools. Large source pools included Dagunguo (Dgg), Gongxiaoshe (Gxs), and Jinze (Jz) springs. Small, high discharge springs included Jiemeiquan (Jmq) and Gumingquan (Gmq). Shallow acidic pools included the spring Zhenzhuquan (Zzq) which exhibited strong outgassing, and a series of pools at Diretiyanqu (Drty). In addition, other types of springs were also present. For example, Huitaijing (Htj) was comprised of two small open wells, with cobble bottoms and relatively low discharge; Shuirebaozha (Srbz) was a shallow spring with a variety of geothermal sources.

Hierarchical clustering divided the spring water chemistry into three well-defined groups based on geographical location (distance) and pH (Figure S2). The neutral-alkaline springs in Rehai (W3) have been previously categorized as Na-Cl-HCO_3_ or Na-HCO_3_-Cl or springs that directly discharge from a high temperature subterranean reservoir within granite or granitic clastic sedimentary host rocks [32], [33]. In contrast, those in Ruidian (W1) have been categorized as Na-HCO_3_ springs [32], [33] and were characterized by high Mg^2+^ and Ca^2+^ concentrations (Table S1), likely caused by dissolution of carbonates. The four acidic sites in Rehai (W2), Zhenzhuquan and Diretiyanqu-1, -2, and -3, exhibited elevated levels of SO_4_^2−^, Mn^2+^, Fe^2+^, Mg^2+^, DOC, and TN (Tables S1 & S2). These data suggest that these acidic springs likely represent vapor condensate-dominated systems that are acidified by oxidation of sulfide to sulfuric acid. The hydrogen and oxygen isotope compositions (Figure S3) were plotted along the local meteoric water line, suggesting that meteoric water was the main source for all springs, as has been previously reported [34]. A large spread in δ^2^H and δ^18^O values for Rehai springs suggest that there were different extents of evaporation, consistent with their different temperatures and varying ratios of surface area/volume. The δ^2^H and δ^18^O values for Ruidian springs were more negative than those for Rehai springs, suggesting Ruidian springs underwent less evaporation, likely due to their moderate temperatures and low ratios of surface area/volume.

Hierarchical clustering of the sediment geochemistry revealed two broad clusters (referred to as S1 and S2) that corresponded to the two geothermal fields (Figure S4). Springs in Ruidian were distinct from those in Rehai in terms of both TOC/TN (51.4% cumulative contribution) and mineralogy (48.6% cumulative contribution). TOC content in the Ruidian spring sediments was generally higher than in the Rehai sediments (Table S1). Silicate minerals dominated the Rehai spring sediments, whereas carbonates (aragonite and calcite) were main minerals in the Ruidian sediments. The solid-phase and aqueous geochemistry of the springs was generally consistent with a granite-hosted system in Rehai and a carbonate-hosted system in Ruidian.

### Microbial Diversity

The two primer sets did not result in any significant differences in microbial diversity (Figure S5) and structure (Figures S6 and S7, Table S3). However, more archaeal sequences were retrieved by the modified primer set than by the original primer set (Table S4). For this reason, the modified primer pair was used for all the samples. A total of 345,802 reads were obtained for 37 samples with the modified primer set after removal of low-quality and chimeric sequences. A variety of taxa were observed at the 97% OTU level, with 21–123 observed and 50–223 predicted OTUs (based on Chao1) and coverage values ranging from 38.2% to 69.9% (Table 2). In Rehai, richness, Shannon diversity, and equitability were not significantly different between sediments and water communities except for Dagunguo, where these indices were higher for sediment samples (Table 2). Similar to Dagunguo, microbial community diversity and richness were much higher in Ruidian sediments than in waters (Table 2). Richness was slightly higher in neutral-alkaline springs than in acidic springs, but Shannon diversity and equitability showed no significant correlation with temperature and pH (Figure S8), or any other selected geochemical variables, which was also confirmed by Mantel tests with all p>0.1. Microbial richness, equitability, and Shannon diversity in Ruidian sinters and sediments were markedly higher than in Rehai sediments (Table 2), and this difference was related to TOC content in sediments/sinters (Chao1 and TOC: r  = 0.42, p<0.05; Shannon diversity and TOC: r  = 0.30, p<0.1).

**Table 2:** Alpha diversity indices at the 97% OTU level of 16S rRNA gene fragments by re-sampling 968 reads in each sample for 1000 replicates with the modified primer set.

Sample ID	Observed OTUs	Coverage^4^ of the observed OTUs (%)	Chao1	Shannon’s diversity	Equitability
Dgg.Water	21	41.90	50	0.77	0.18
Dgg.TFF	24	40.70	59	0.88	0.19
Dgg^1^.Sinter1	72	58.90	122	2.75	0.45
Dgg.Sinter2	90	47.10	191	3.33	0.51
Dgg.Sinter3	93	59.44	156	3.48	0.53
Drty-3.Sediment	46	69.89	66	3.04	0.55
Drty-2.Water	39	59.14	65	2.13	0.40
Drty-2.Sediment	46	64.20	71	2.61	0.47
Drty-1.Water	49	56.74	87	1.88	0.33
Drty-1.Sediment	43	53.40	81	1.19	0.22
GmqP.Water	58	40.79	143	1.92	0.33
GmqP.Sediment	35	41.97	83	1.24	0.24
GmqP.Streamer	30	41.94	73	1.18	0.24
GmqC.Water	102	45.80	224	2.98	0.45
GmqC.Sediment	52	48.71	108	2.54	0.45
GmqS.Water	82	56.70	145	2.85	0.45
JmqL.Water	59	41.03	143	2.27	0.39
JmqL.Sediment	74	54.03	138	3.33	0.53
JmqR.Water	62	38.24	161	2.46	0.41
JmqR.Sediment	42	47.26	88	1.69	0.31
Zzq.Water	46	49.98	93	1.88	0.34
HtjR.Water	63	49.28	128	2.79	0.47
SrbzU.Water	87	55.38	157	3.00	0.46
SrbzU.Sediment	62	47.08	132	2.42	0.41
SrbzD.Sediment	86	52.61	164	3.50	0.54
GxsS.Water	41	61.76	67	1.84	0.34
GxsS^2^.Sintert2	121	54.17	223	4.90	0.71
GxsS.Sinter3	121	61.30	198	4.80	0.69
GxsB.Water	37	64.58	57	1.73	0.33
GxsB^3^.Sediment1	123	59.27	207	5.09	0.73
GxsB.Sediment2	112	57.88	193	4.72	0.69
GxsB.Sediment3	110	56.36	195	4.78	0.71
Jz.Water	40	46.15	86	1.32	0.25

1 Sinter samples from different locations within the source pool of Dagunguo.

2 Sinter samples from different sites on the edge of the source pool of Gongxiaoshe.

3 Sediment and sinter samples from different sites on the bottom of the source pool of Gongxiaoshe.

4 Coverage is the ratio of the observed OTUs to Chao1.

Sample datasets that contained less than 968 reads were excluded from this table.

### Overall Microbial Community Structure in Relation to Geochemistry

The results of pairwise comparisons using ANOSIM suggested that there was no significant difference in microbial community structure either between Rehai and Ruidian springs or between water and sediment, with the R-statistic value of 0.16 and 0.02, respectively, and p>0.05 for both comparisons. When the geothermal field and the sample type were considered together, the test results showed that microbial community structure in Ruidian sediment was distinctly different from those of the other three groups: Ruidian water, Rehai water, and Rehai sediment with the R-statistic value of 0.71, 0.28, and 0.41, respectively and p<0.05 (for all 3 comparisons). Addition of the pH factor in the analysis further divided the Rehai samples to two distinct groups: low (2.5–4.8) and high (6.7–9.4) pH springs (all R-statistic values near 1 and p<0.01); and Ruidian water samples were similar to Rehai high pH samples in microbial composition. In summary, three distinct groups were identified based on ANOSIM: 1) Ruidian sediment; 2) Rehai low pH, and 3) Rehai high pH samples (both sediment and water) and Ruidian water samples.

**Figure 2:**
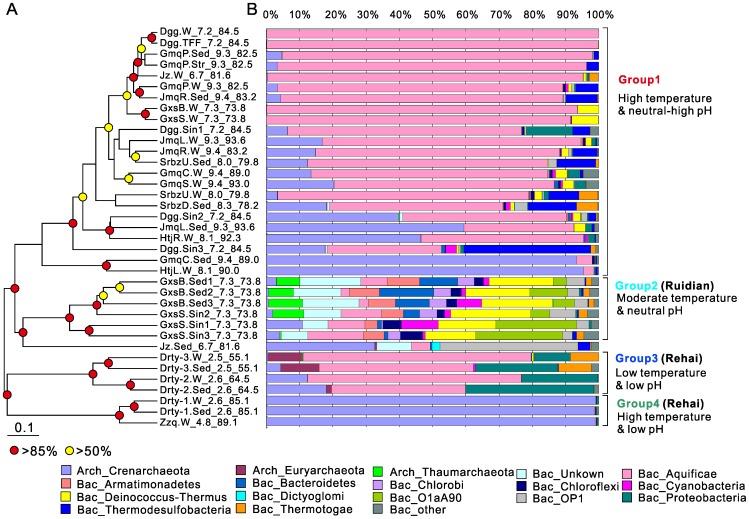
Microbial community composition grouped by pH and temperature. A. UPGMA cluster tree based on Bray-Curtis dissimilarity obtained based on the 97% cutoff level; **B.** microbial compositions at the phylum level. Filled circles at nodes represent jackknife values. “TFF”, “Sin”, “Sed”, “Str” and “W” in sample ID refer to the tangential flow filtration (TFF) sample (only from Dagunguo), sinter, sediment, streamer and water samples, respectively. The numbers in the site name are the pH and temperature of each site. The prefix “Bac” denotes bacterial phyla, and the prefix “Arch” denotes archaeal phyla. Only the microbial groups with abundance higher than 2% are displayed. The groups with abundances lower than 2% are included as “Others”.

A similar grouping pattern was also identified based on Bray-Curtis dissimilarity at the 97% similarity OTU level using a UPGMA cluster tree (Figure 2). Four groups were identified that could be differentiated based on the various combinations of the geothermal field, pH, and temperature (T). The four groups were: Group 1: high T and neutral to alkaline pH (namely Rehai high pH water+sediment and Ruidian water samples); Group 2: moderate T and neutral pH (Ruidian sediments); Group 3: low T and low pH (Rehai); and Group 4: high T and low pH (Rehai). These groups were similar to those identified by the ANOSIM analysis. Mantel tests revealed that community structure was significantly correlated with pH and temperature (r = 0.47 and 0.44, respectively; p  = 0.001). The BIO-ENV results confirmed that community structure was significantly correlated with a combination of pH and temperature (r  = 0.73).

To reveal associations between microbial composition and geochemistry, geochemical groups were plotted along with the four microbial groups for a direct visual comparison (Figure 1). In general, microbial community structure broadly corresponded to water and sediment geochemistry but with some exceptions. For example, Jiemeiquan, Gumingquan, Huitaijing, Shuirebaozha, and Dagunguo exhibited similar aqueous geochemistry and similar water-borne microbial communities. Likewise, Shuirebaozha, Gumingquan, and Jiemeiquan exhibited similar sediment geochemistry and similar benthic microbial composition, suggesting that sediment geochemistry is important in shaping microbial community structure in sediment. However, in some cases, geochemistry and microbial community structure did not correspond to each other. For example, despite having similar sediment geochemistry to Jiemeiquan, Gumingquan, and Shuirebaozha, the acidic hot springs in Rehai (Drty-1, -2, and -3) had a different benthic community.

### Microbial Community Composition in Relation to Geochemistry

**Figure 3:**
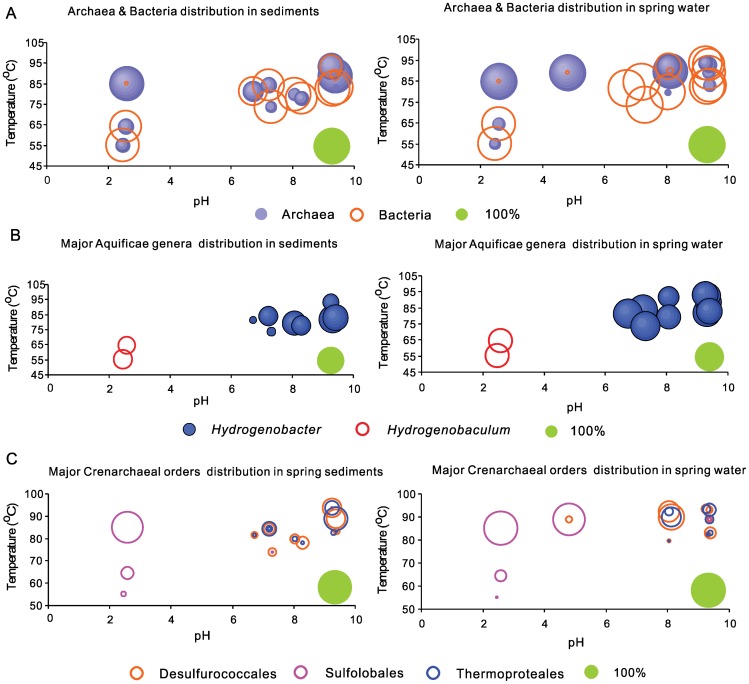
Percentages of different microbial groups in sediments (left panels) and spring water (right panels) from Tengchong. The size of the green circle on each plot serves as a scale and represents 100% of abundnace. **A.** Archaeal and Bacterial distributions: higher percentage of Archaea tended to occur in springs with higher temperature and lower pH. **B.** Aquificae genera distributions: distributions of *Hydrogenobacter* spp. and *Hydrogenobaculum* spp.; *Hydrogenobacter*-related sequences were abundant in neutral or alkaline springs, whereas *Hydrogenobaculum*-related sequences were abundant in acidic springs. **C.** Crenarcaheal order distributions: *Desulfurococcales* and *Thermoproteales* within class *Thermopotei* of phylum *Crenarchaeota* were abundant in neutral-alkaline springs, whereas *Sulfolobales* within class *Thermopotei* of phylum *Crenarchaeota* was abundant in acidic springs. These two groups did not co-exist in the same spring.

Average archaeal abundance was much higher in Rehai springs (31±35%) than in Ruidian springs (9±10%). In some high temperature acidic (i.e., Diretiyanqu-1 and Zhenzhuquan) and alkaline (Gumingquan and Huitaijing) springs in Rehai, more than 90% of total sequences in the pyrotag dataset was composed of archaea (Figure 2B, Figure 3A). To reveal differences in specific taxonomic groups between Rehai and Ruidian as well as among the springs of a given region, SIMPER analysis was used to identify the top ten taxonomic groups that contributed to the dissimilarities or similarities among the selected groups of hot springs (Table 3). At the regional scale, there were differences at the family/genus level between Rehai and Ruidian (Table 3, 1^st^ comparison), *Hydrogenobacter* was present in both Rehai and Ruidian, but its relative abundance was higher in Rehai. *Sulfolobus, Desulfurococcaceae, Hydrogenobaculum, Pyrobaculum,* and *Thermodesulfobacteriaceae* were present in Rehai, but not in Ruidian. Likewise, unclassified bacteria, candidate phylum O1aA90 and OP1, *Thermaceae*, and *Rhodothermaceae* were significantly more abundant in Ruidian. Several of the thermoacidophilic taxa were present only in Rehai because of the presence of acidic springs; therefore, acidic springs were removed from the analysis and the comparison between Rehai and Ruidian was repeated (Table 3, 2^nd^ comparison). This revealed that *Hydrogenobacter*, *Desulfurococcaceae*, *Pyrobaculum*, and *Thermodesulfobacteriaceae* were more abundant in Rehai, whereas *Thermus*, yet-uncultivated groups O1aA90, *Thermaceae*, *Rhodothermaceae*, and a yet-unnamed novel bacterial group were more abundant in Ruidian.

**Table 3:** Top ten OTUs (at the 97% level) responsible for dissimilarity between certain selected groups of hot spring samples.

Rehai (A) vs. Ruidian (B)								
Taxon^1^	Family/Genus		Contrib.^2^ (%)			Avg3.abund.(A) (%)		Avg.abund.(B) (%)
Aquificae	Hydrogenobacter		30.85			43.41		34.11
Thermoprotei	Sulfolobus		6.61			22.96		0.00
Thermoprotei	Desulfurococcaceae		4.08			4.68		0.00
Aquificae	Hydrogenobaculum		3.58			2.83		0.00
Thermoprotei	Pyrobaculum		3.06			3.65		0.00
Deinococcus-Thermus^4^	Thermus		2.49			0.46		6.66
Thermodesulfobacteria	Thermodesulfobacteriaceae		2.27			3.05		0.00
O1aA90	Unclassified O1aA90		2.18			0.02		5.78
Deinococcus-Thermus	Thermaceae		1.99			0.03		5.92
Bacteriodetes	Rhodothermaceae		1.91			0.01		7.15
**Neutral-Alkaline Rehai springs (A) vs. Ruidian springs (B)**								
**Taxon**		**Family/Genus**		**Contrib. (%)**		**Avg.abund.(A) (%)**		**Avg.abund.(B) (%)**
Aquificae		Hydrogenobacter		36.77		65.22		34.11
Thermoprotei		Desulfurococcaceae		5.51		20.22		0.00
Thermoprotei		Pyrobaculum		4.14		19.76		0.01
Thermodesulfobacteria		Thermodesulfobacteriaceae		3.05		20.56		0.00
Deinococcus-Thermus		Thermus		2.43		3.91		10.10
O1aA90		Unclassified O1aA90		2.15		0.02		9.77
Deinococcus-Thermus		Thermaceae		1.98		0.04		6.76
Bacteriodetes		Rhodothermaceae		1.91		0.01		8.76
Unidentified Bacteria		Unidentified Bacteria		1.71		0.02		6.12
**High T & neutral-alkaline pH (Rehai only, A) vs. High T & low pH (B)**								
**Taxon**	**Family/Genus**		**Contrib. (%)**			**Avg.abund.(A) (%)**		**Avg.abund.(B) (%)**
Thermoprotei	Sulfolobus		49.18			0.04		82.21
Aquificae	Hydrogenobacter		21.42			64.76		0.11
Thermoprotei	Sulfolobus		3.52			0.00		3.08
Thermoprotei	Desulfurococcaceae		3.23			7.17		0.00
Thermoprotei	Pyrobaculum		2.48			5.59		0.00
Thermodesulfobacteria	Thermodesulfobacteriaceae		1.88			4.66		0.00
Thermoprotei	Sulfolobus		1.71			0.00		2.45
Thermoprotei	Sulfolobus		1.43			0.00		1.79
Thermoprotei	Sulfolobus		1.00			0.00		1.14
Thermoprotei	Pyrodictiaceae		0.75			0.04		0.65
**Low T & low pH (A) vs. High T & low pH (B)**								
**Taxon**	**Family/Genus**			**Contrib. (%)**	**Avg.abund.(A) (%)**		**Avg.abund.(B) (%)**	
Thermoprotei	Sulfolobus			57.70	2.72		82.21	
Aquificae	Hydrogenobaculum			11.61	48.74		0.00	
Alphaproteobacteria	Acidisphaera			5.19	21.81		0.00	
Thermoprotei	Sulfolobus			4.26	0.00		3.08	
Thermoprotei	Sulfolobus			2.05	0.01		2.45	
Thermoprotei	Sulfolobus			1.72	0.63		1.79	
Thermoprotei	Metallosphaera			1.46	6.39		0.02	
Thermoprotei	Sulfolobus			1.23	0.48		1.14	
Deltaproteobacteria	Desulfurella			0.98	3.56		0.00	
Thermoprotei	Pyrodictiaceae			0.89	0.00		0.65	

1 Phylum level for most bacteria and class level for archaea and Proteobacteria.

2 Contribution of each OTU to the overall dissimilarity between these two clusters.

3 Average abundance of each OTU in cluster (a) and cluster (b).

4 *Thermaceae* is a family in the phylum *Deinococcus-Thermus* and includes genus *Thermus*. Whereas some OTUs can be classified to genus level (*Thermus*), others can only be classified to the family *Thermaceae*.

Within Rehai, the bacterial phylum *Aquificae* and the archaeal phylum *Crenarchaeota* (primarily class *Thermoprotei*) were the predominant groups, comprising 72±26% and 92±23% of total bacteria and archaea, respectively (as opposed to 35±41% and 55±46%, respectively within Ruidian). The percentage of *Aquificae* in water samples (71.1% ±31.6%) was higher than in sediments or sinters (33.7% ±28.9%). In addition, the microbial composition within *Aquificae* and *Thermoprotei* varied according to environmental conditions. Within the *Aquificae*, *Hydrogenobacter* was abundant in springs with circumneutral to alkaline pH (6.7–9.4, Table 3, 3^rd^ comparison and Figure 3B), whereas *Hydrogenobaculum* was the dominant *Aquificae* genus in low temperature, acidic, and sulfur-rich springs (T: 55.1–64.5°C, pH: 2.5–2.6; Table 3, 4^th^ comparison and Figure 3B); however, it was not present in high temperature acidic sites (T: 85.1–89.1°C and pH: 2.6–4.8). Similarly, the three orders of *Thermoprotei, Sulfolobales, Desulfurococcales* and *Thermoproteales*, showed distinctive distribution patterns. *Sulfolobales*, predominantly the genus *Sulfolobus*, was dominant in high temperature, acidic, and sulfur-rich springs (T: 85.1–89.1°C and pH: 2.5–4.8), comprising >82% of all sequences (Table 3, 3^rd^ and 4^th^ comparisons; Figures 2B & 3C). In contrast, *Desulfurococcales* and *Thermoproteales* (mainly *Pyrobaculum*) were the dominant archaea in springs with circumneutral to alkaline pH (pH: 6.7–9.4, Figure 3C) and high concentrations of silica, Na, K and Cl (Table 1). Within acidic sites, temperature exerted a strong control on community composition. With decreased temperature, a *Sulfolobus*-dominated community gave way to a more diverse community with a relatively high abundance of the bacterial taxa *Hydrogenobaculum*, *Acidisphaera*, and *Desulfurella*, and the archaeal taxon *Metallosphaera* (Table 3, 4^th^ comparison). The relative abundance of *Sulfolobus* increased linearly with increased temperature in the acidic samples (Pearson correlation = 0.97, p = 0.002).

Some bacterial and archaeal groups were abundant only in specific samples. For example, *Thermaceae* (*Deinococcus-Thermus* phylum) and *Rhodothermaceae* (*Bacteriodetes* phylum) were fairly abundant only in samples from Gongxiaoshe in Ruidian. *Thermodesulfobacteriaceae* were prominent in several Rehai springs with high temperature (78.2–93.6°C) and neutral-alkaline pH (7.2–9.4) (Figure 2 and Table 3). Putative ammonia-oxidizing archaea in the *Thaumarchaeota* (mainly related to “*Candidatus* Nitrosocaldus”) were the dominant archaea in Gongxiaoshe spring (74% of total archaea, which was ∼10% of total sequences in the dataset), whereas the order *Thermoplasmatales* in the *Euryarchaeota* were the predominant archaea in the lowest temperature acidic site, Diretiyanqu 3.

## Discussion

### Correlation between Microbial Diversity and Geochemistry

Markedly higher microbial richness, equitability, and diversity in Ruidian sinters and sediments than in sediments and waters from Rehai (Table 2) may be due to the circumneutral pH, moderate temperatures, and high TOC contents of the Ruidian springs relative to those from Rehai (Table 1). Different mineralogy between Ruidian and Rehai sediments/sinters (carbonates vs. silicates) may have contributed to the difference in microbial diversity between these two hydrothermal fields as well. However, because of the similarly low diversity in both Ruidian and Rehai waters, it is likely that sediment properties (i.e., high TOC, carbonate mineralogy) may be more important in accounting for the high diversity in Ruidian sediments and sinters. A previous study also revealed a much higher microbial diversity in sediment than in water of Great Boiling Spring in Nevada, which may be related to long water residence time [20]. Ruidian springs were expected to have long resident time and may have accounted for the large difference in diversity between the sediment and water.

### Comparison with Previous Studies in Tengchong Hot Springs

Previous studies of thermophiles from Tengchong springs have mostly focused on microbial cultivation studies [8]. Our molecular examination of the springs detected many of the organisms previously isolated, but these taxa tended to be sub-dominant. For example, the bacterial genera *Meiothermus* and *Thermus* and the archaeal genera *Acidianus* and *Metallosphaera* have been previously isolated from Rehai springs and were detected, but not abundant in our study. Notably, our study revealed the dominance of *Sulfolobus* in high temperature, acidic, and high-sulfur springs in Rehai and a diversity of *Sulfolobales* isolates from Tengchong have been described previously [35]. However, many microorganisms, such as *Firmicutes*, that have been isolated from Tengchong springs were not detected in this study. Although many of the dominant taxa in circumneutral and alkaline springs in Tengchong have close relatives in culture, few, if any, of those organisms have been isolated from Tengchong springs. This discrepancy is particularly clear among the *Aquificae* and the crenarchaeal orders *Desulfurococcales* and *Thermoproteales*.

A comparison between the previous clone library-based studies and this study reveals consistency between the compositions of the community. Orders *Sulfolobales, Desulfococcales*, and *Thermoproteales* were all identified in our study, consistent with the results reported for Tengchong springs using a specific primer set for *Crenarchaeota* [36]. A recent clone library-based study showed that both bacterial phylum *Aquificae* and archaeal phylum *Crenarchaeota* were detected in a microbial mat sample from Tengchong (near Shuirebaozha) [11], consistent with our results. In contrast, sequences related to putative AOA were not detected in Rehai springs, despite their reported presence using a functional gene approach and the presence of lipid biomarkers (e.g., crenarchaeol) proposed to be diagnostic of AOA [13], [14], [37]. This discrepancy is likely due to the lower temperatures of the springs previously sampled in Tengchong [13] and is consistent with the low *amoA* transcript levels detected [14] in some of the spring that were also sampled in this study (i.e., Dagunguo, Diretiyanqu, Zhenzhuquan). It was likely that the abundance of AOA population was low in these springs.

### Microbial Community Composition in Tengchong in Comparison to other Geothermal Systems

The composition of microbial communities in Tengchong appeared to be dependent on geothermal region, spring type, residence time, and geochemical conditions. For large source pools with standing water (Dagunguo, Gongxiaoshe, and Jinze), there were substantial differences in microbial community composition between water and sediment. The estimated long water residence times in these springs may allow a genuine planktonic community to develop [20]. For small source pools with high discharge, there was no significant difference between water and sediment, likely because of their dynamic mixing.

Microbial community composition and its correlation with geochemistry in Tengchong can be better understood by a comparison to other geothermal systems in the world. The hot spring systems of YNP and the Great Basin are well-studied examples for this purpose. In terms of mechanisms and genesis, the Tengchong hot springs are more similar to those in YNP in that they are both volcanically driven with acidic springs. In contrast, geothermal activity in the Great Basin is attributed to tectonically driven dilation of range-front faults and there are no highly acidic springs (e.g., pH <5.5).

Both Tengchong and YNP host springs with dramatically different geochemical conditions and both display a bi-modal pH distribution due to buffering by either the carbonate system or the sulfuric acid system [38]. As a result, many springs in Rehai and YNP harbor similar microbial communities at the phylum and family/genus levels. Among abundant microbial groups in Tengchong and YNP are the bacteria phyla *Aquificae, Deinococcus-Thermus, Thermodesulfobacteria*, and the archaeal phylum *Crenarchaeota*.

#### Aquificae

Four genera of *Aquificales*, *Thermocrinis*, *Sulfurihydrogenibium*, *Hydrogenobacter* and *Hydrogenobaculum*, are common in terrestrial geothermal environments and have been previously observed in YNP [39]–[44]. All these genera were present in Tengchong springs; however, their relative abundance was somewhat surprising. *Hydrogenobacter* and *Hydrogenobaculum* were abundant in high temperature (73.8–93.6°C), circumneutral to alkaline pH (6.7–9.4) springs and low temperature (55.1–64.5°C), acidic (pH: 2.5–2.6), sulfur-rich springs, respectively; however, *Thermocrinis* and *Sulfurihydrogenibium* were apparently present at very low relative abundance (<0.02% of all reads). *Thermocrinis* and *Hydrogenobacter* usually occupy similar niches in high temperature, circumneutral to alkaline (∼pH 8) terrestrial systems, although *Hydrogenobacter* may be more abundant at lower temperatures (60–80°C) than *Thermocrinis* (75–92°C) and is not known to form macroscopically visible streamers [39], [45]. Given the high temperatures sampled here (>78.2°C) and the abundance of streamer biomass in alkaline spring sources (pH >8.3) in Rehai, it is surprising that *Hydrogenobacter* would dominate over *Thermocrinis*. However, a recent study [44] has shown that *Thermocrinis*-dominated *Aquificales* are present in both streamer biofilm community (SBC) and non-SBC locations of YNP, and casted doubt on the hypotheses that *Aquificales* (especially *Thermocrinis*) are truly responsible for streamer formation. Our data in Tengchong supported this observation: microbial composition of the streamer sample from Gumingquan was nearly the same as that in the Gumingquan sediment without streamer, consisting of predominantly *Aquificae* with small amounts of *Thermodesulfobacteria* and *Crenarchaeota* (*Desulfurococcales*) (Fig. 2B). In Tengchong, *Hydrogenobacter* was the dominant *Aquificae* in both streamer-forming and non-streamer forming communities. At present, it remains unclear about the mechanisms of streamer formation, although it was possible that some other organisms may be working with *Aquificae* to either encourage or suppress streamer formation [44].

*Sulfurihydrogenibium* is common and abundant in circumneutral springs in YNP [46]–[49] and the Great Basin [20]. Its apparent low abundance in Tengchong springs may be related to their low maximum growth temperature of 78°C, which was below the lowest temperature in the circumneutral and alkaline samples reported here except Ruidian springs (Gongxiaoshe and Jinze). The Ruidian springs, however, were expected to have a relatively high dissolved O_2_ content due to the long water residence time, which may be incompatible with the microaerophilic nature of *Sulfurihydrogenibium* (optimal O_2_ concentration, 6% v/v) [50].

#### Deinococcus-Thermus

Although many isolates from this phylum have been obtained from Rehai [8], our molecular study showed that this group was actually the most abundant in Gongxiaoshe of Ruidian, which is cooler and more oxygenated than circumneutral and alkaline springs sampled in Rehai. *Thermus* has a similar geochemical distribution in YNP [40], [44], [48], [51] and the Great Basin [52], [53] as in Tengchong. The distributions of these organisms in Tengchong, YNP, and the Great Basin are consistent with the physiology of cultured representatives within this phylum [54].

#### Thermodesulfobacteria

*Thermodesulfobacteria* were found in spring sediments with neutral pH (7.2) and high temperature (84.5°C), consistent with their common presence in sediments of YNP [40], [41], [44], [46] and the Great Basin [52], [53] under the conditions of near neutral pH (6.1–7.3) and high temperature (77–90°C). The presence of these organisms in sediments is also consistent with their physiology, which is either anaerobic sulfate or ferric iron reduction.

#### Crenarchaeota

The crenarchaeal order *Thermoprotei* was abundant in Rehai and also at many sites in YNP [41], [44], [47], but only a small component in the Great Basin, where *Archaeoglobales*, *Thaumarchaeota*, and yet-uncultivated lineages were more abundant [20], [52], [53]. This difference is likely due to the fact that there are no highly acidic springs in the Great Basin (pH <5.5) and therefore cannot support the many members of the order *Thermoprotei* that are generally acidophilic.

The relative proportions of three orders within this class, *Sulfolobales, Thermoproteales*, and *Desulfurococcales*, correlated with pH and temperature (Figure 3C), and other geochemical conditions in Tengchong springs. The predominance of *Sulfolobales* in high temperature, acidic, sulfur-rich sites (Diretiyanqu-1, -2, and -3) in both water and sediment (well mixed with water), is consistent with the oxygen, temperature and pH optima of the genus *Sulfolobus* (i.e., microaerophilic, 65–85°C and 2–3) and the distribution of *Sulfolobus* in acidic sulfataras in YNP and elsewhere [55], [56]. The dominance of *Sulfolobus* at these springs with high levels of sulfur (primarily sulfate) and ferrous iron suggests that aerobic respiration of sulfide, Fe^2+^, organic matter, and H_2_ are dominant metabolisms in high temperature acid springs in Rehai [55]. In addition to the genus *Sulfolobus* of the family Sulfolobaceae of the order *Sulfolobales*, the genera *Acidianus* and *Metallosphaera* were also present in low abundance yet neither *Stygiolobus* nor *Sulfurisphaera* were detected. *Stygiolobus*-like sequences were present in YNP springs with dissolved oxygen levels at or below detection [43], consistent with the anaerobic nature of this genus [55]. The possible absence of *Stygiolobus* in Tengchong springs may be due to the oxic nature of these springs.

The predominance of *Desulfurococcales* in Rehai springs with high temperature (>80°C) and circumneutral to alkaline pH (Figure 3C) is consistent with the known hyperthermophily of these organisms (temperature optima 84–95°C; [57]), but only partly overlaps with the known pH range of known terrestrial genera of *Desulfurococcales* (pH range 2.2–8.5; [57]) and their pH distribution in YNP springs based on cultivation-independent studies (pH range 6.1–8.1; [41], [43], [44]. Thus, it appears that *Desulfurococcales* in Tengchong have a higher pH range than other terrestrial *Desulfurococcales*, and even higher than the marine organism *Aeropyrum pernix*, which is able to grow up to pH 9.0 [57]. Interestingly, the two Rehai springs where abundant *Desulfurococcales* were observed, i.e., Gumingquan and Jiemeiquan, also had high levels of Si, Cl, K, and Na in the waters (Table S1). At present, it is unclear if these ions affected the pH tolerance of *Desulfurococcales*. The higher abundance of *Desulfurococcales* in the sediments of Gumingquan and Jiemeiquan than those in the corresponding waters (Fig. 3C) may suggest that the sediments may have offered some protection against highly alkaline pH.

Similar to *Desulfurococcales*, *Thermoproteales* (mainly *Pyrobaculum*) relatives were the most abundant in Rehai springs with high temperature and neutral-alkaline pH (Table 3, Figure 3C). Again the high temperature of these springs is consistent with the hyperthermophilic nature of the genus *Pyrobaculum* (74–102°C with optimum 100°C, [58]), but the pH of some Rehai springs is higher than both the pH optimum (6–7) and even the maximum (9) known for this genus [58]. Thus, the *Pyrobaculum* in Rehai are likely to be more alkaliphilic than any known *Thermoproteales* including those from YNP [47], [59]. Within the order *Thermoproteales*, the terrestrial genera *Thermofilum*, *Vulcanisaeta*, *Thermoproteus*, and *Thermocladium* are commonly observed in YNP springs with mildly acidic pH (∼5.5–6.1) and moderate to high temperature [41], [46], [47], which is consistent with the physiology of these genera. The apparent absence of these genera in Tengchong appears to be consistent with the paucity of springs in this pH range.

### Physiological Functions

Although it can be difficult to infer physiological functions based on DNA sequences, especially for partial sequences, there are a few taxonomic groups for which tentative functions might be inferred. As expected, phototrophic bacteria were only detected in the Gongxiaoshe spring, which is consistent with the observation that the upper temperature limit for photosynthesis is 73–75°C [38], [60]. pH was also a limiting factor as no prokaryotic phototrophs were observed in acidic springs even when temperatures were lower than 73°C, as consistent with a previous study [61].

Sulfide or Fe^2+^ oxidation with O_2_ as an electron acceptor appears to be an important metabolic pathway in acidic springs of Rehai (Diretiyanqu and Zhenzhuquan, mostly by *Sulfolobus*), as it is in YNP [62], [63]. For neutral and alkaline hot springs, members of the *Aquificales* were prominent in springs of Rehai (Tengchong), as they are in many springs in YNP (e.g. [39], [40], [42]–[44], indicating that chemolithotrophy by oxidation of H_2_, reduced sulfur compounds (thiosulfate, sulfur, or sulfide), or one-carbon compounds (formate or formaldehyde) are likely important metabolic processes in these springs.

The majority of archaeal sequences in Gongxiaoshe were closely related (>97%) to “*Candidatus* Nitrosocaldus yellowstonii”, a known AOA [64] within the phylum *Thaumarchaeota* [65], suggesting a potentially important role for ammonia oxidation in this spring. AOA are also prominent members that have been observed in Great Boiling Spring of the Great Basin [52] and in hydrothermal vent features in Yellowstone Lake [29]. Interestingly, springs from these three different regions share broadly similar pH and temperature conditions, i.e., circumneutral pH (6.2–8.4) and moderate temperature (60–82°C). These conditions are similar to, but extend above, the temperature range of “*Candidatus* Nitrosocaldus yellowstonii” in enrichment culture [64].

### Conclusions

Two geothermal fields in Tengchong, Rehai and Ruidian, were highly diverse in their environmental conditions, encompassing a temperature range from 55.1 to 93.6°C and pH from 2.46 to 9.39 as well as wide ranges of water and sediment geochemistry. The overall microbial diversity was low, and did not show any systematic correlation with geochemical conditions. Ruidian sediments hosted higher microbial diversity than the corresponding waters and Rehai samples, likely due to mild temperature, pH, high organic carbon, and different mineralogy (carbonate-dominated).

The bacterial phylum *Aquificae* and archaeal phylum *Crenarchaeota* were dominant in all samples from Rehai and water samples from Ruidian. The specific compositions of *Aquificae* and *Crenarchaeota* were highly dependent on pH, temperature, and water/sediment geochemistry. Acidic and sulfur-rich springs were dominated by crenarchaeal order *Sulfolobales* and *Aquificae* genus *Hydrogenobaculumn*, whereas neutral to alkaline pH favored the *Aquificae* genus *Hydrogenobacter*, and crenarchaeal orders *Desulfurococcales* and *Thermoproteales*. A higher pH tolerance was observed for *Desulfurococcales* and *Thermoproteales* and this tolerance appeared to be related to water/sediment geochemistry. Whereas many of these organisms have been detected in other terrestrial springs worldwide, some members typically found in other terrestrial hot springs such as *Thermocrinis* were not present or only in minor abundance in Tengchong. This study highlights the importance of temperature, pH and other geochemical conditions and endemism in shaping the microbial community structure in Tengchong hot springs.

## Supporting Information

Figure S1 **Field photos of Tengchong hot springs. A.** Dagunguo (Dgg), the largest spring at Rehai, is a ∼5 meter diameter, 1.5 meter deep, cylindrical pool with vigorously degassing sources (84.5°C). This clear, circumneutral chloride spring has hard silica precipitates. Sinter samples were chipped from the sides of the spring in three locations; **B.** Diretiyanqu (Drty) at Rehai is comprised small acid sulfate pools (pH 2.5, labeled B, C, D) derived from vapor condensate. Springs vary with respect to temperature (55.1–85.1°C) but all are turbid with high dissolved clay content. Pools and fumaroles are temporally variable; **C.** Gumingquan is the most alkaline spring in Rehai (pH 9.35). The high discharge source pictured at left (GmqS, 93°C) has several small downstream pools. GmqC is a large pool (89°C) near the center of the flow path (photo at right). GmqP, the largest pool (82.5°C), is located immediately below a bridge (photo at center) and contained abundant streamer biomass at the time of sampling; **D.** Jiemeiquan (also known as Yanjingquan) at Rehai includes a pair of ∼1 m diameter, high temperature, alkaline springs (pH 9.3). Left Zimeiquan (JmqL, labeled H) has an extremely active 93.6°C source. Right Zimeiquan (JmqR, labeled J) has a cooler (83.2°C) and less active source; **E.** Zhenzhuquan (Zzq) at Rehai is a shallow, heart-shaped, acidic spring (pH 4.79) with several vigorously degassing sources (89.1°C); **F.** Huaitaijing at Rehai includes two 1 m diameter wells, left (HtjL, 90°C labeled L) and right (HtjR, 92.3°C, labeled M). Both wells are approximately pH 8.1 and have large boulder bottoms, which precluded sediment sampling; **G.** Shuirebaozha at Rehai is a shallow pool with a bottom of soft clay and small stones with several degassing sources. SrbzU (79.8°C, pH 8.04 labeled N) is a peninsula between two pools, and SrbzD (78.2°C, pH 8.28, labeled P) is located near the middle of the west pool; **H.** Gongxiaoshe is a large, circumneutral, carbonate-depositing spring in Ruidian. Samples were collected at temperatures of 73.8°C and included sediments (GxsB) from the bottom of the pool and sinter (GxsS) chipped from the sides of the spring; **I.** Jinze (Jz) is one of many geothermal wells in Ruidian. Sediment samples were collected from the bottom of the well at pH 6.71 and 81.6°C.
(DOC)

Figure S2 **Hierarchical clusters of Euclidean distance for water geochemistry.** The geochemical data include TOC, TN, cation/anion concentrations, and trace metal concentrations. The numbers in the site name are the pH and temperature of each site. The scale bar on the top represents Euclidean distance.
(TIF)

Figure S3 **The δ^2^H (‰) - δ^18^O (‰) relationship for hot spring waters from Ruidian and Rehai.** This relationship is compared to the global meteoric water line (GMWL) [66] and local meteoric water line (LMWL). The LMWL is developed based on precipitation data at the Xishuangbanna station, about 400 km south of Tengchong [67].
(DOCX)

Figure S4 **Hierarchical clusters of Euclidean distance for sediment/sinter geochemistry**. The clustering is based on TOC, TN, and mineral compositions in sediment/sinter. The scale bar on the top represents Euclidean distance.
(TIF)

Figure S5 **Comparison of diversity indices at the 97% cutoff level between microbial communities retrieved with the two primer pairs.**
(TIF)

Figure S6 **Unweighted UniFrac cluster tree based on microbial communities at the 97% cutoff level.** The letter “P” at the end of the sample ID refers to a microbial community retrieved with the unmodified primer pair.
(TIF)

Figure S7 **Comparison of microbial compositions retrieved with the two primer pairs.**
(DOC)

Figure S8 **Diversity indices of different microbial communities as a function of temperature and pH.** The red circle on the lower right side of each plot is an indicator of scale. For example, for Chao 1, the size of the red circle represents Chao 1 value of 100. By comparing with the size of the red circle, all values of diversity indices can be determined.
(TIF)

Table S1 **Water and sediment geochemistry for Tengchong springs.**
(DOCX)

Table S2 **Trace metal concentrations of hot spring water samples from Tengchong (values in ppb).**
(DOCX)

Table S3 **Significance tests of the overall microbial community structure between the two primer sets with three different statistical approaches.**
(DOCX)

Table S4 **Comparison of microbial communities retrieved with the two primer sets.**
(DOCX)
