# Supplementary figures and images for: A Comprehensive Census of Microbial Diversity in Hot Springs of Tengchong, Yunnan Province China Using 16S rRNA Gene Pyrosequencing
Source: PLoS One. 2013 Jan 9;8(1):e53350. doi: 10.1371/journal.pone.0053350 (PMC3541193; doi:10.1371/journal.pone.0053350)

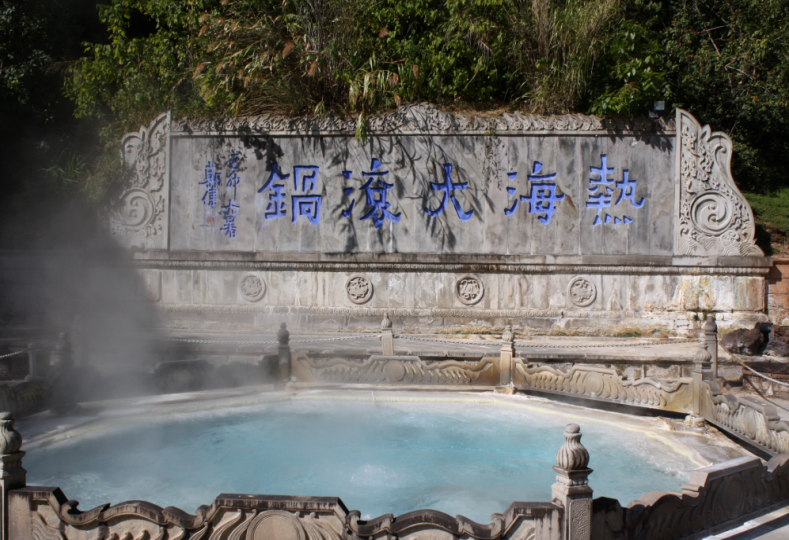


**Figure S1-A**


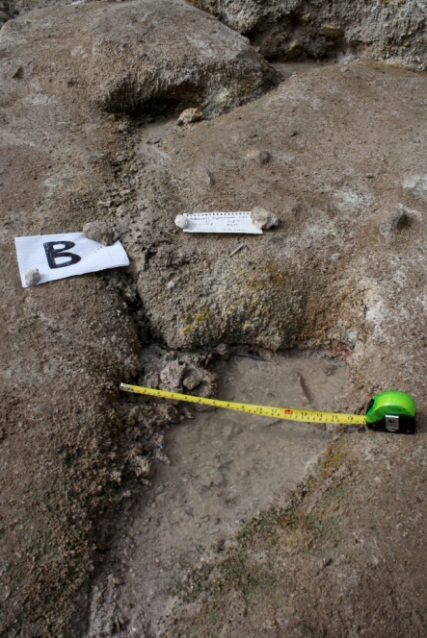

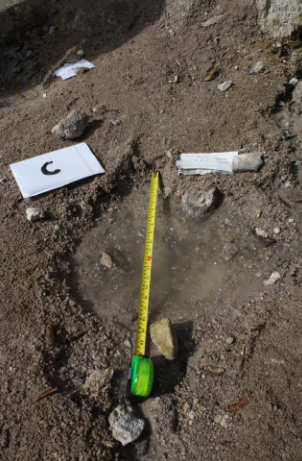

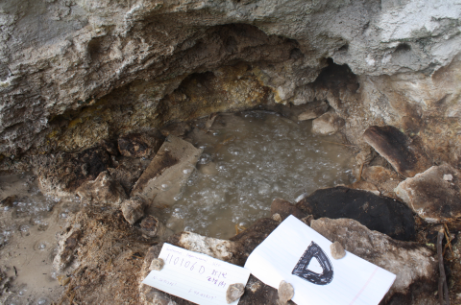


**Figure S1-B**


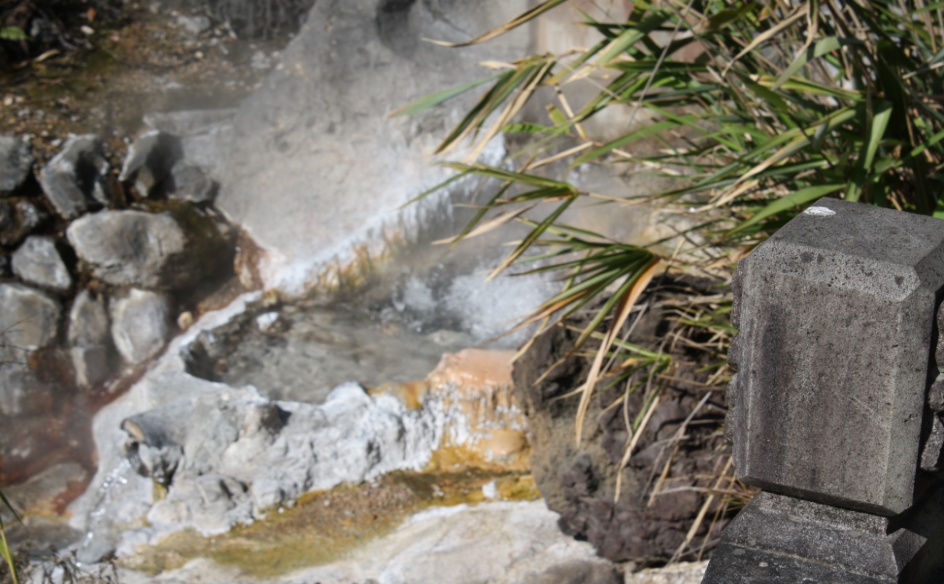

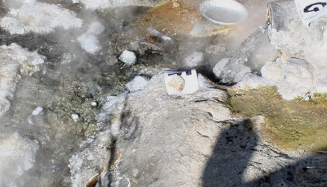

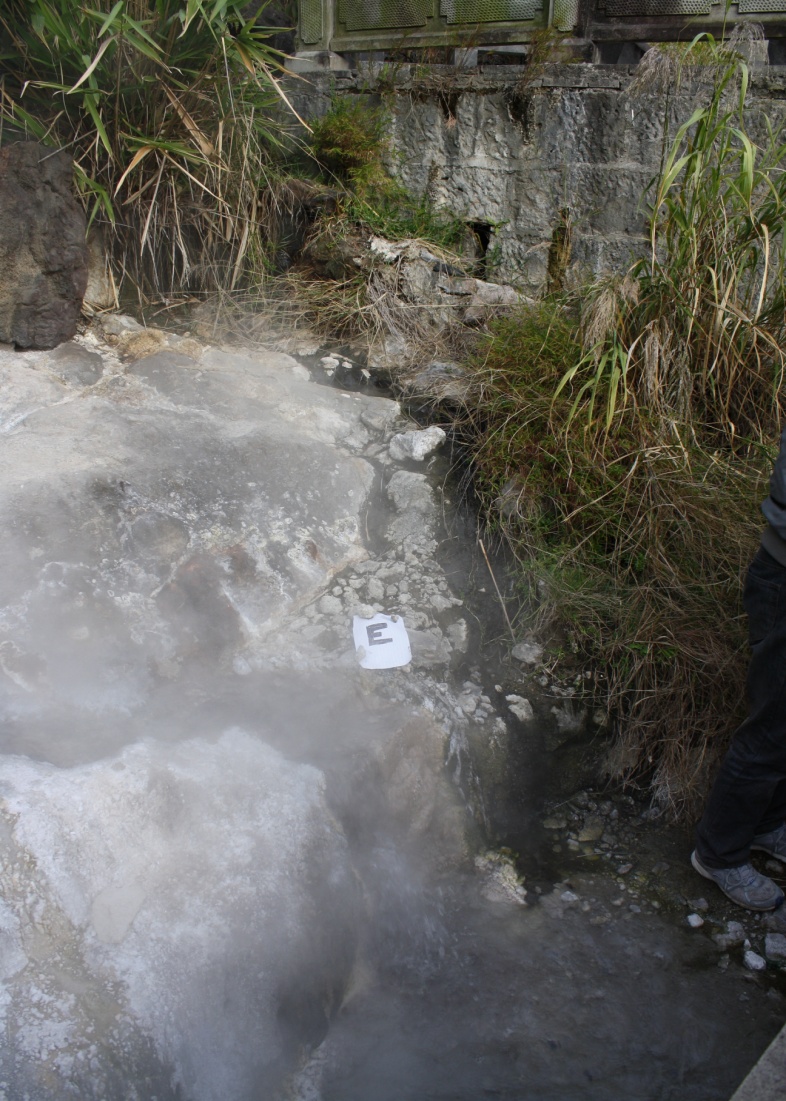


**Figure S1-C**


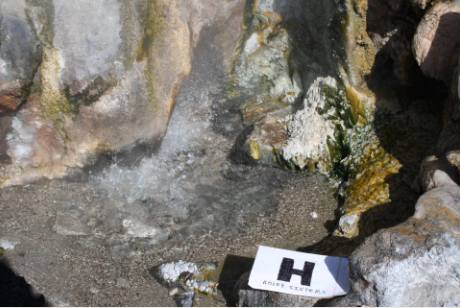

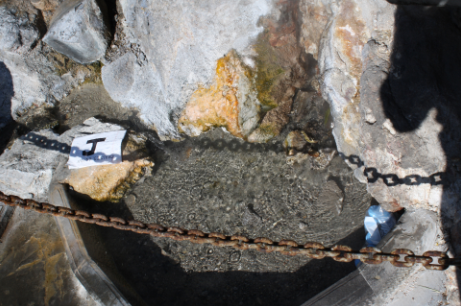


**Figure S1-D**


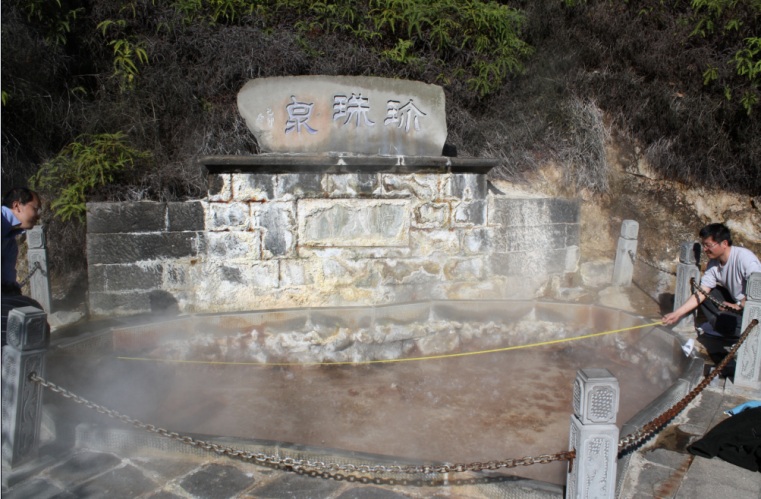
**Figure S1-E**


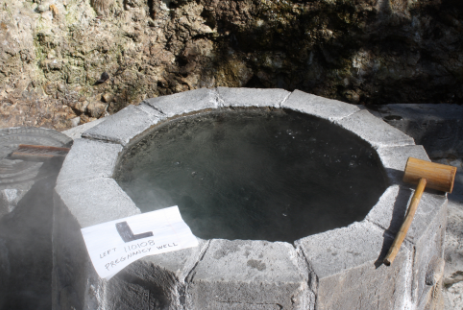

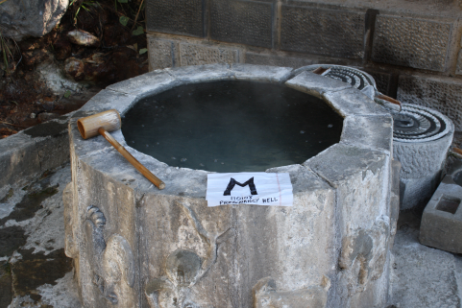


**Figure S1-F**


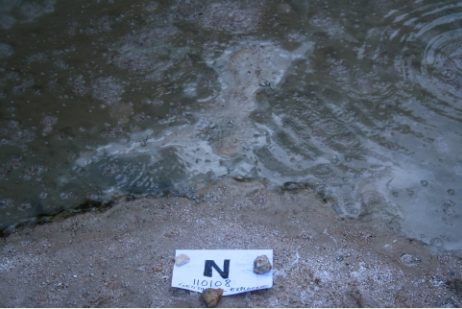


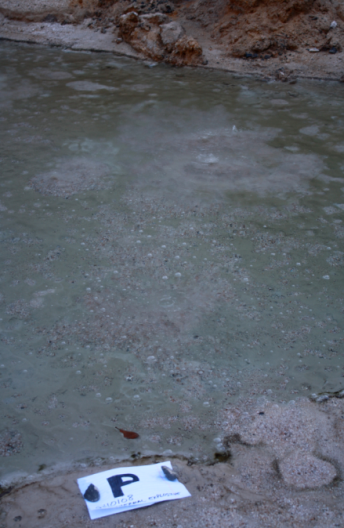


**Figure S1-G**


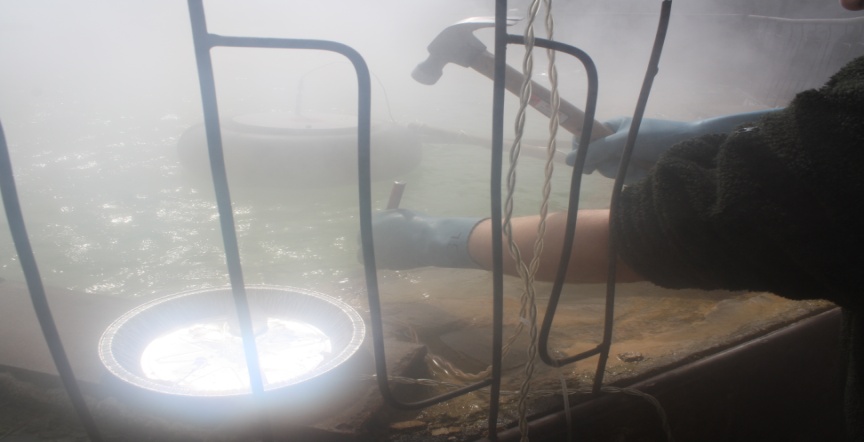

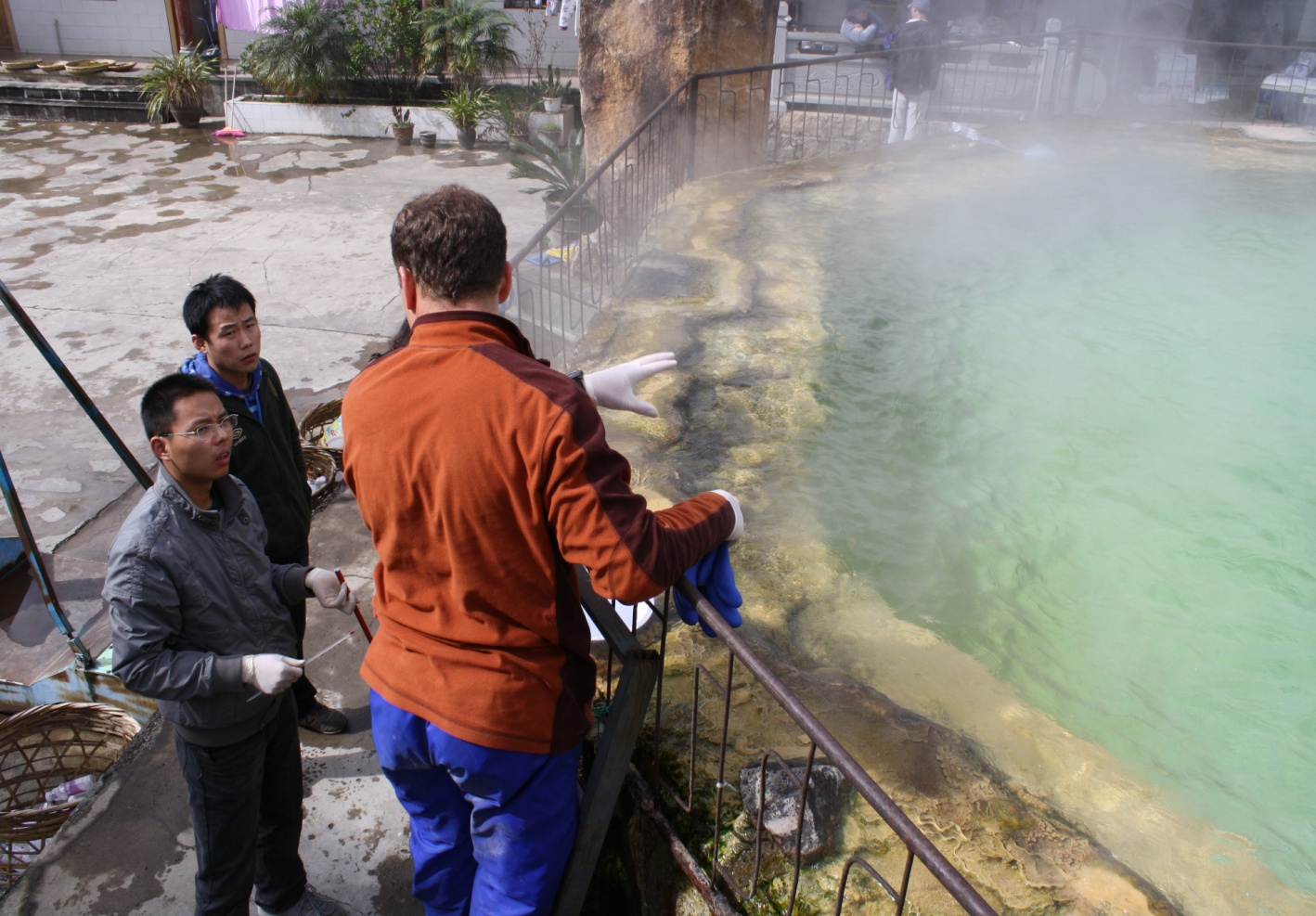

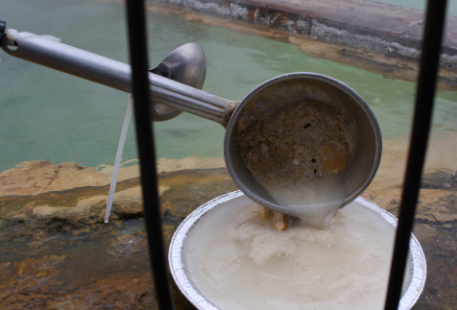


**Figure S1-H**

**
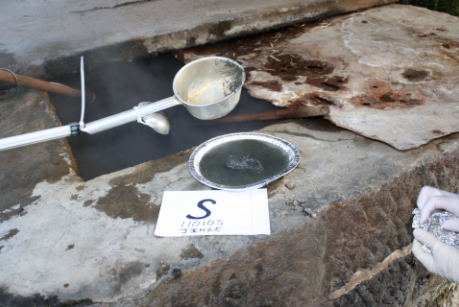
**

**Figure S1-I**

Supplement: Figure S1 — Field photos of Tengchong hot springs. A. Dagunguo (Dgg), the largest spring at Rehai, is a ∼5 meter diameter, 1.5 meter deep, cylindrical pool with vigorously degassing sources (84.5°C). This clear, circumneutral chloride spring has hard silica precipitates. Sinter samples were chipped from the sides of the spring in three locations; B. Diretiyanqu (Drty) at Rehai is comprised small acid sulfate pools (pH 2.5, labeled B, C, D) derived from vapor condensate. Springs vary with respect to temperature (55.1–85.1°C) but all are turbid with high dissolved clay content. Pools and fumaroles are temporally variable; C. Gumingquan is the most alkaline spring in Rehai (pH 9.35). The high discharge source pictured at left (GmqS, 93°C) has several small downstream pools. GmqC is a large pool (89°C) near the center of the flow path (photo at right). GmqP, the largest pool (82.5°C), is located immediately below a bridge (photo at center) and contained abundant streamer biomass at the time of sampling; D. Jiemeiquan (also known as Yanjingquan) at Rehai includes a pair of ∼1 m diameter, high temperature, alkaline springs (pH 9.3). Left Zimeiquan (JmqL, labeled H) has an extremely active 93.6°C source. Right Zimeiquan (JmqR, labeled J) has a cooler (83.2°C) and less active source; E. Zhenzhuquan (Zzq) at Rehai is a shallow, heart-shaped, acidic spring (pH 4.79) with several vigorously degassing sources (89.1°C); F. Huaitaijing at Rehai includes two 1 m diameter wells, left (HtjL, 90°C labeled L) and right (HtjR, 92.3°C, labeled M). Both wells are approximately pH 8.1 and have large boulder bottoms, which precluded sediment sampling; G. Shuirebaozha at Rehai is a shallow pool with a bottom of soft clay and small stones with several degassing sources. SrbzU (79.8°C, pH 8.04 labeled N) is a peninsula between two pools, and SrbzD (78.2°C, pH 8.28, labeled P) is located near the middle of the west pool; H. Gongxiaoshe is a large, circumneutral, carbonate-depositing spring in Ruidian. Sam [file pone.0053350.s001.doc]

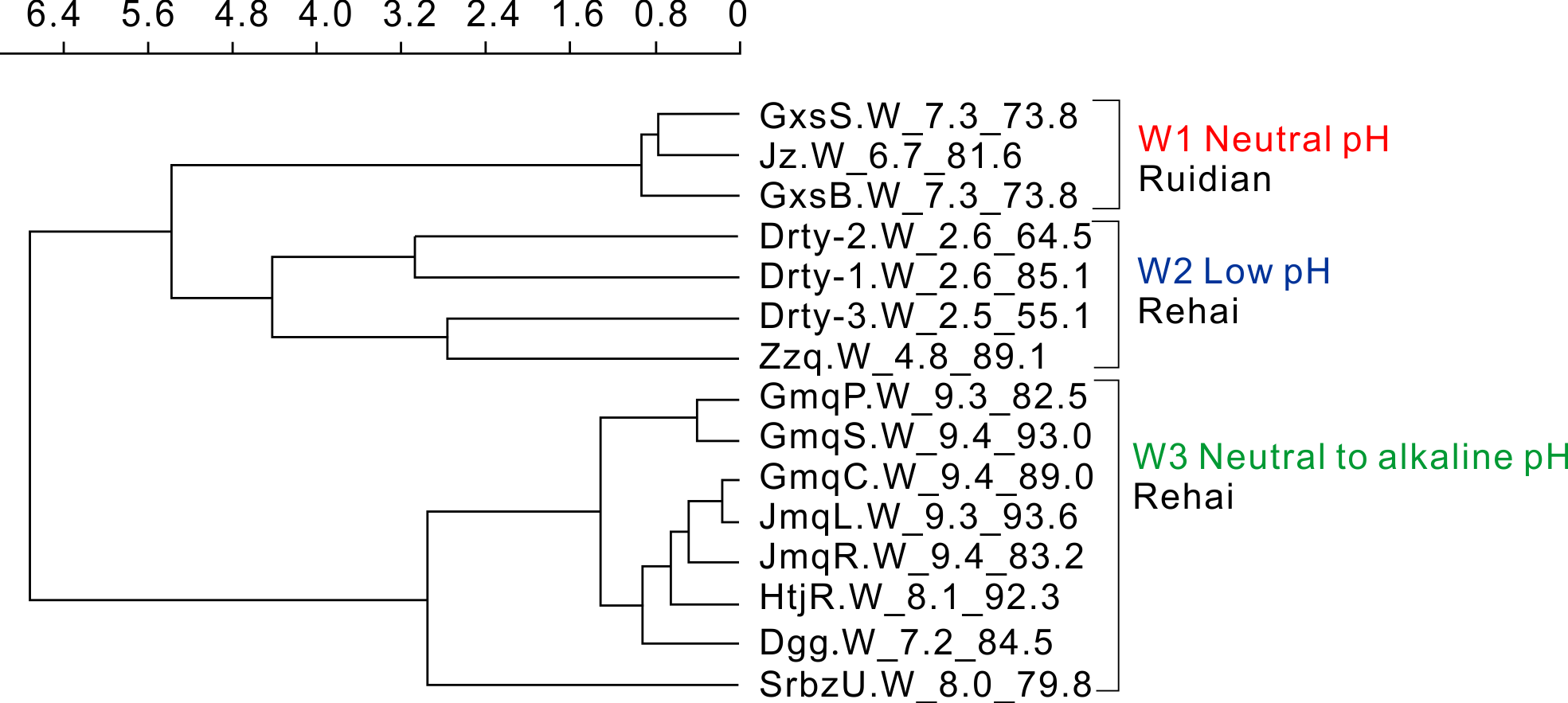

Supplement: Figure S2 — Hierarchical clusters of Euclidean distance for water geochemistry. The geochemical data include TOC, TN, cation/anion concentrations, and trace metal concentrations. The numbers in the site name are the pH and temperature of each site. The scale bar on the top represents Euclidean distance. (TIF) [file pone.0053350.s002.tif]

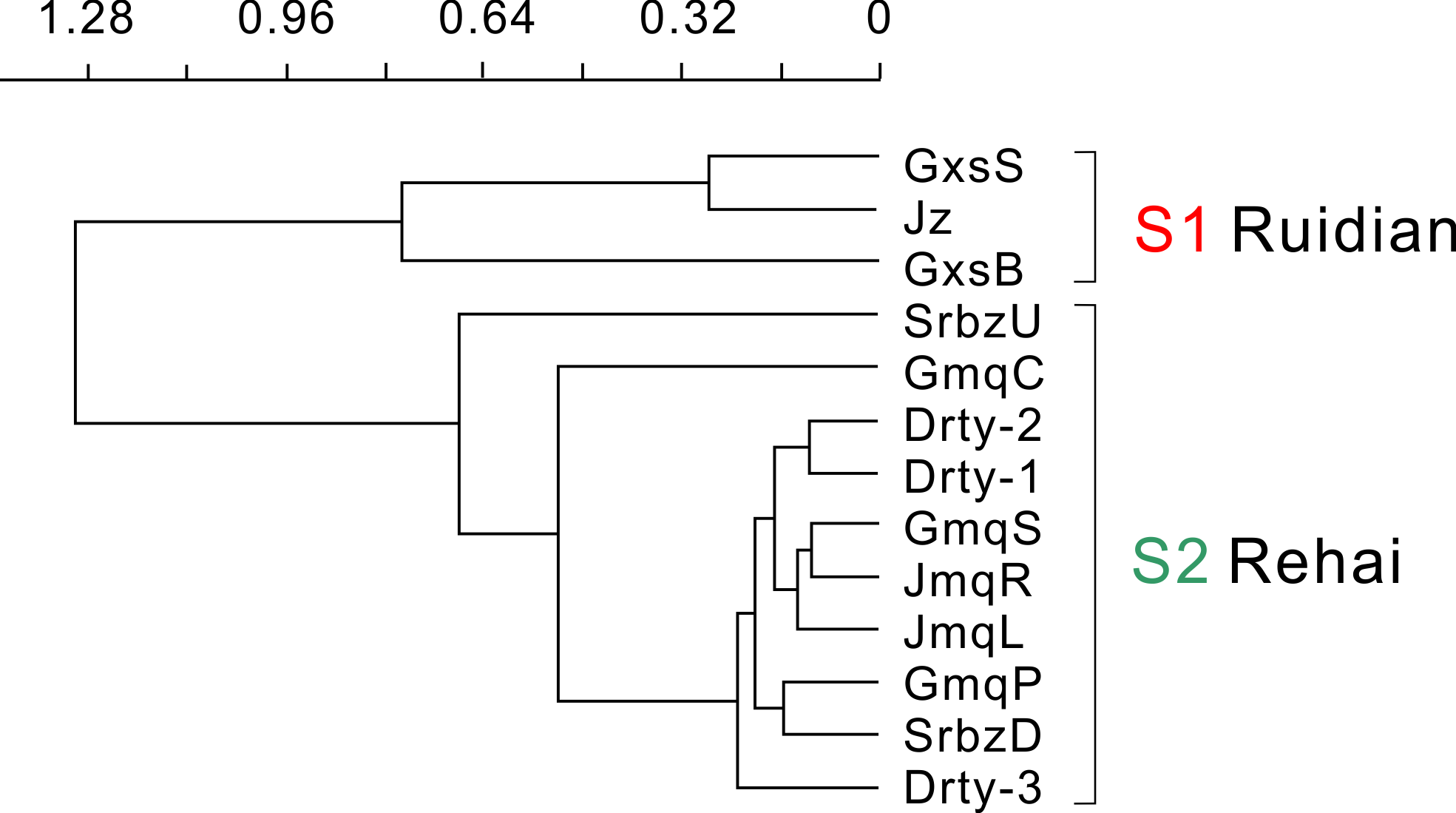

Supplement: Figure S4 — Hierarchical clusters of Euclidean distance for sediment/sinter geochemistry. The clustering is based on TOC, TN, and mineral compositions in sediment/sinter. The scale bar on the top represents Euclidean distance. (TIF) [file pone.0053350.s004.tif]

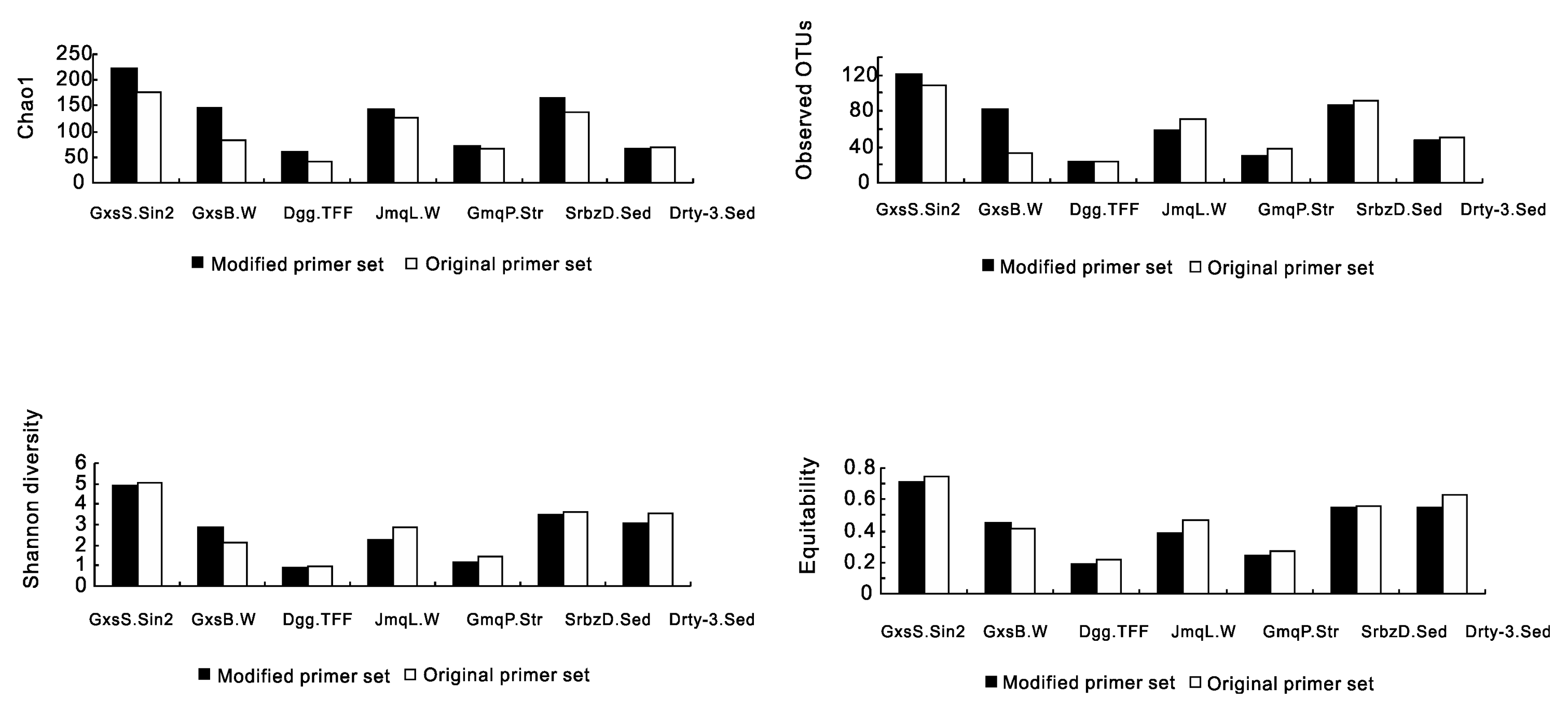

Supplement: Figure S5 — Comparison of diversity indices at the 97% cutoff level between microbial communities retrieved with the two primer pairs. (TIF) [file pone.0053350.s005.tif]

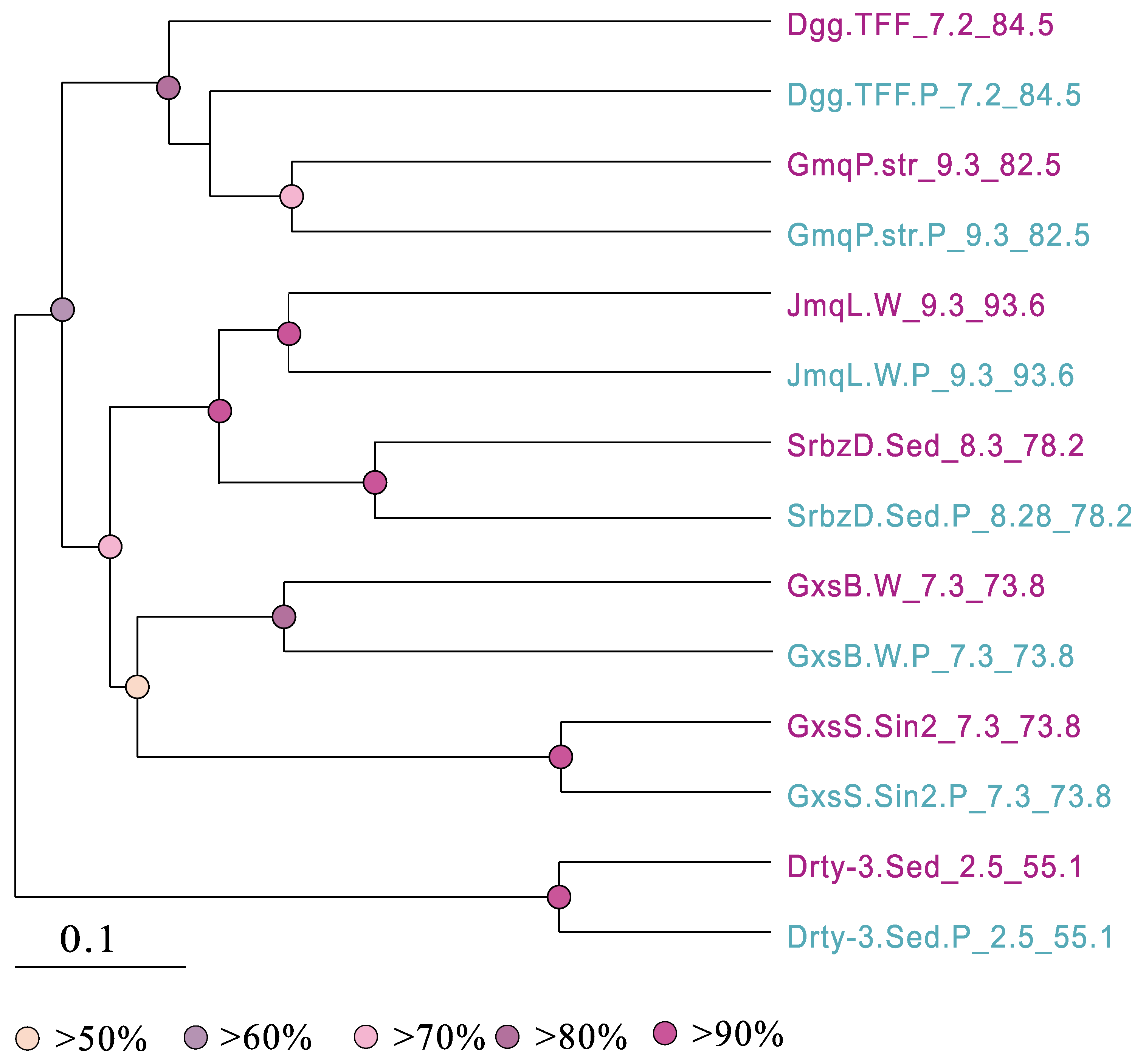

Supplement: Figure S6 — Unweighted UniFrac cluster tree based on microbial communities at the 97% cutoff level. The letter “P” at the end of the sample ID refers to a microbial community retrieved with the unmodified primer pair. (TIF) [file pone.0053350.s006.tif]

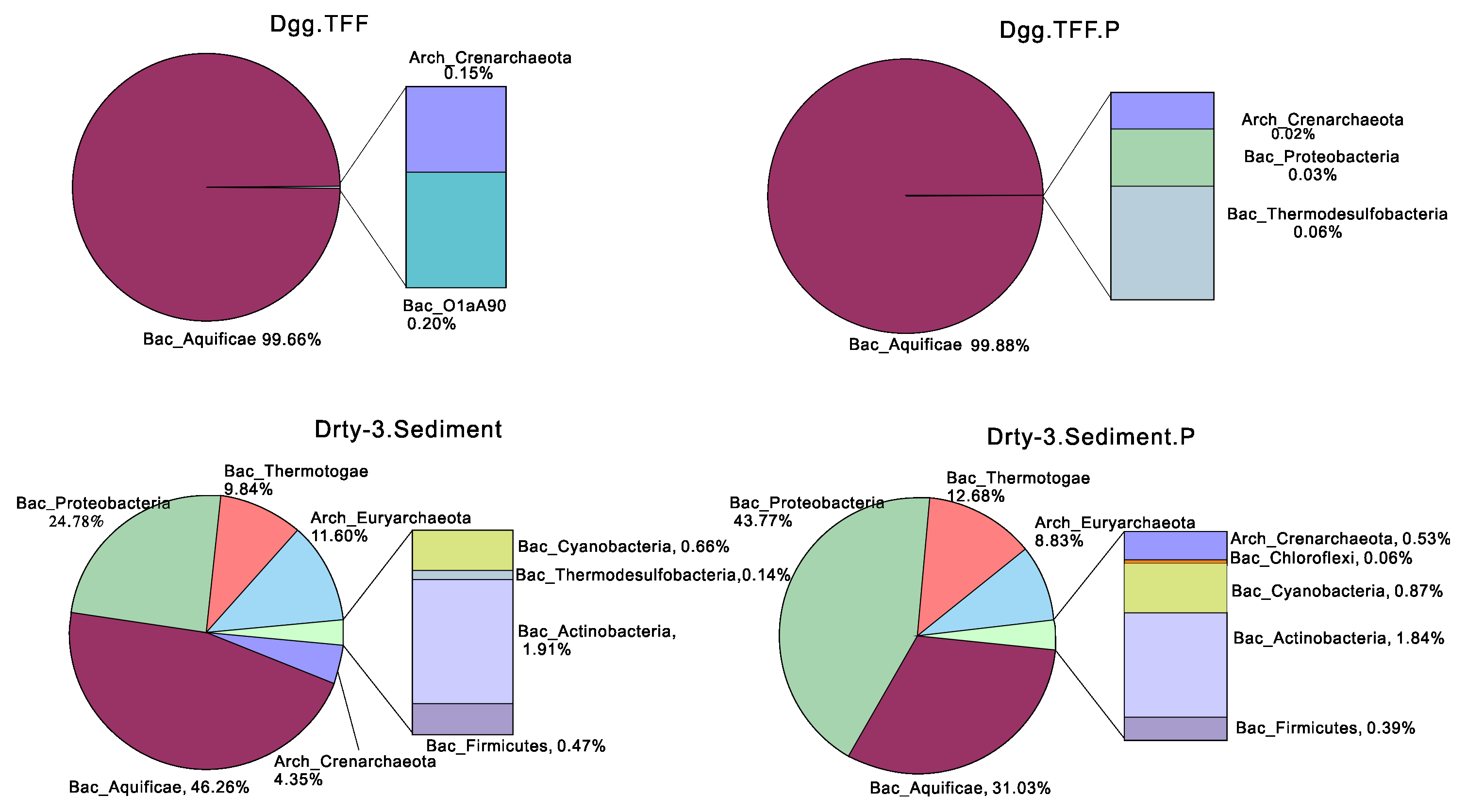


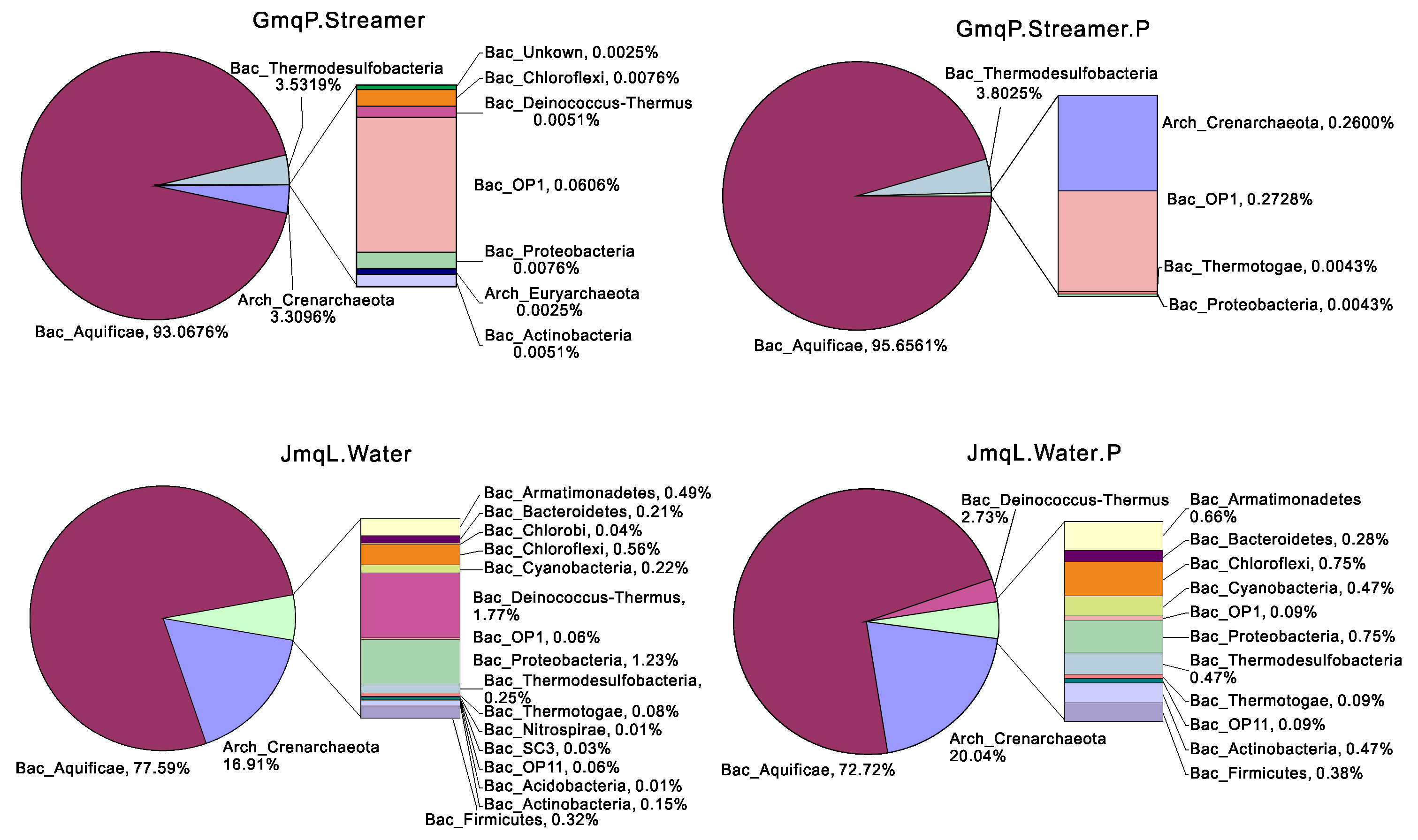


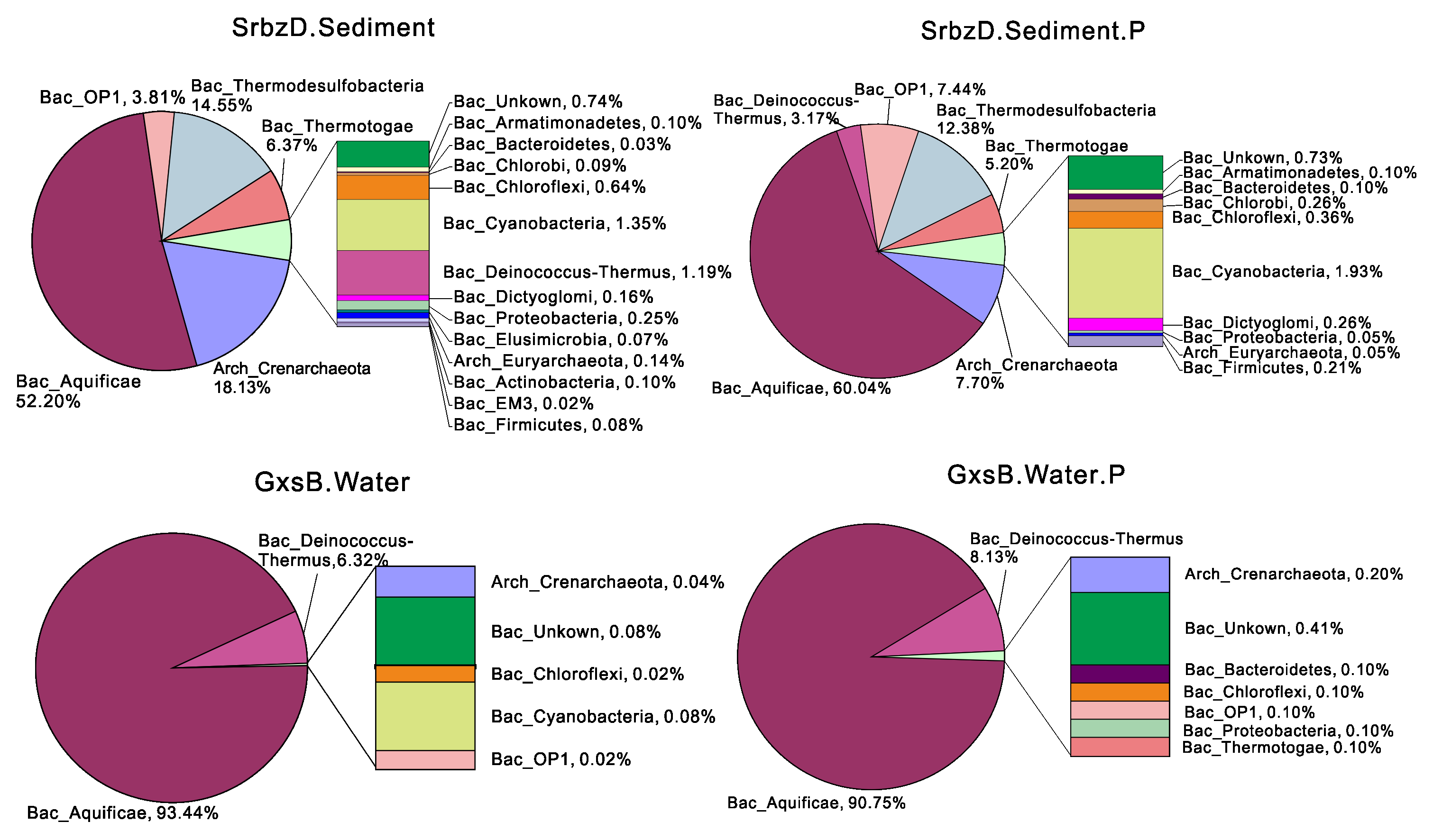


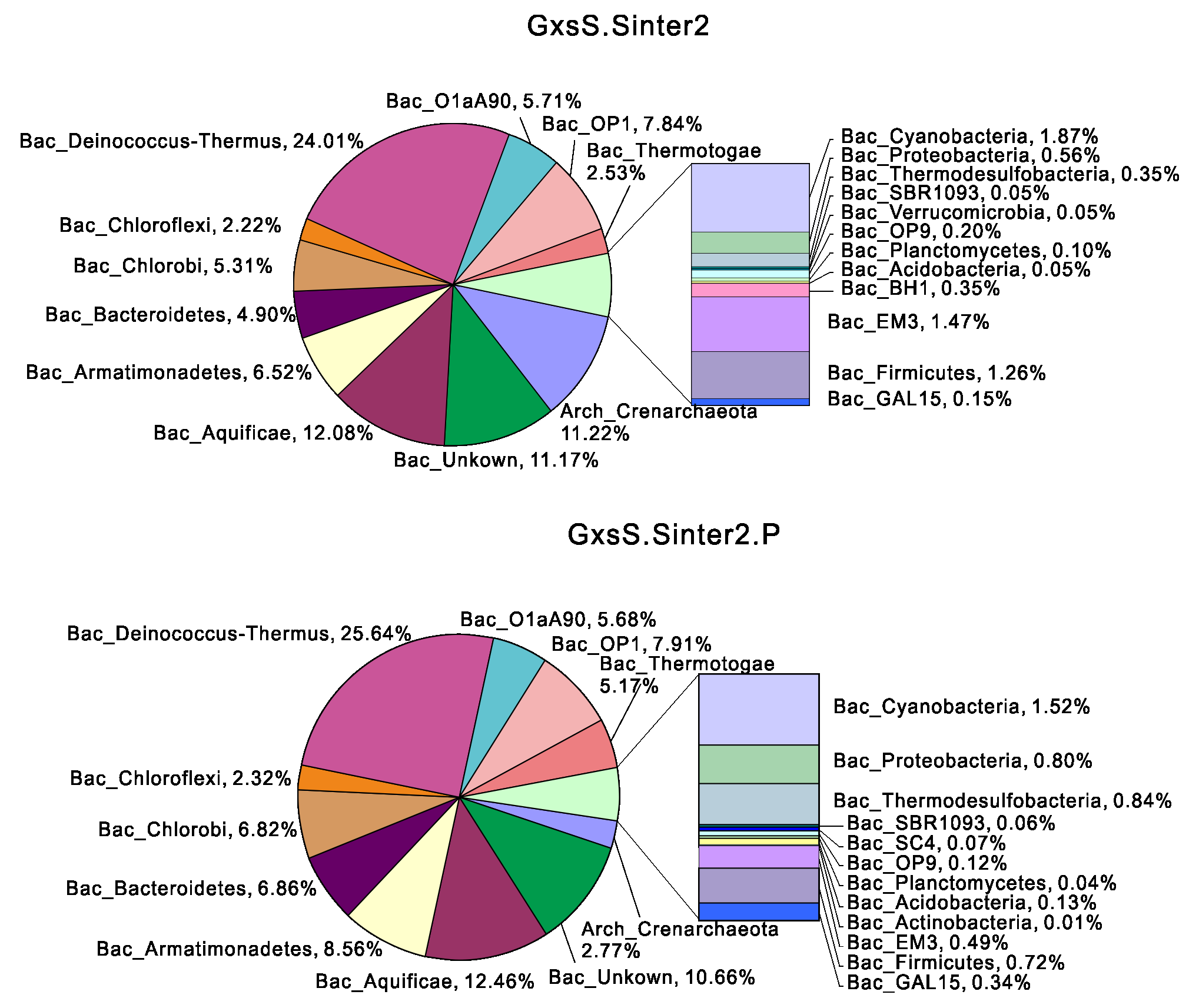

Supplement: Figure S7 — Comparison of microbial compositions retrieved with the two primer pairs. (DOC) [file pone.0053350.s007.doc]

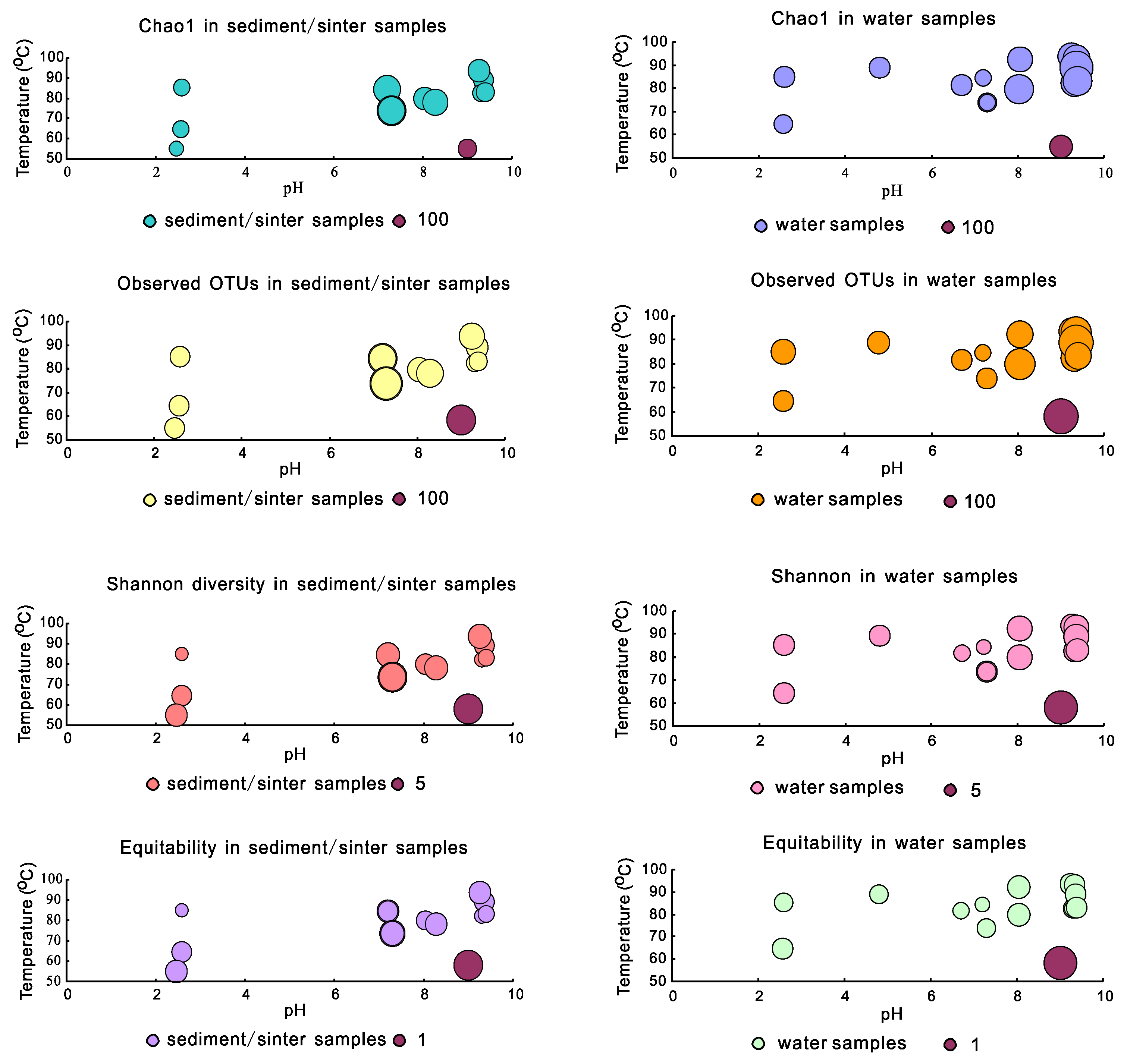

Supplement: Figure S8 — Diversity indices of different microbial communities as a function of temperature and pH. The red circle on the lower right side of each plot is an indicator of scale. For example, for Chao 1, the size of the red circle represents Chao 1 value of 100. By comparing with the size of the red circle, all values of diversity indices can be determined. (TIF) [file pone.0053350.s008.tif]
