# Supplementary material for: A Comprehensive Census of Microbial Diversity in Hot Springs of Tengchong, Yunnan Province China Using 16S rRNA Gene Pyrosequencing
Source: PLoS One. 2013 Jan 9;8(1):e53350. doi: 10.1371/journal.pone.0053350 (PMC3541193; doi:10.1371/journal.pone.0053350)
Supplement: Table S1 — Water and sediment geochemistry for Tengchong springs. (DOCX) [file pone.0053350.s009.docx]

**Table S1** Water and sediment geochemistry for Tengchong springs^*^

|  | | Rehai thermal field | | | | | | | | | | | | | | | Ruidian thermal field | | |
| --- | --- | --- | --- | --- | --- | --- | --- | --- | --- | --- | --- | --- | --- | --- | --- | --- | --- | --- | --- |
| Sample code^1^ | | Dgg | Drty-1 | Drty-2 | Drty-3 | GmqS | GmqC | GmqP | JmqL | JmqR | Zzq | HtjL | HtjR | SrbzU | SrbzD | | GxsS | GxsB | Jz |
| Field parameter measured with probes | Conductivity (mS/cm) | 5.1 | 1.5 | 2.0 | 2.0 | 4.0 | 4.0 | 3.9 | 4.1 | 3.8 | 0.4 | 3.8 | 3.5 | 2.8 | 2.7 | 2.8 | | 2.8 | 2.2 |
| Field parameters measured with colorimetry | S ^2-^ (ug/L) | 0.1 | 0.8 | bdl | 0.2 | 0.1 | 0.3 | 0.2 | 0.2 | 0.2 | 0.2 | 0.1 | 0.1 | bdl | bdl | bdl | | bdl | bdl |
|  | NH_4_^+^ ( mg/L) | 0.06 | bdl | 0.11 | bdl | bdl | bdl | bdl | bdl | bdl | bdl | 0.25 | bdl | bdl | 0.15 | bdl | | 0.1 | bdl |
|  | NO_2_^-^ + NO_3_^-^ ( mg/L) | 2.3 | 3.6 | 1.2 | 2.6 | 1.5 | 1.9 | 2.2 | 1.7 | 1.4 | bdl | 2.9 | 2.4 | 1.2 | 1.9 | 0.7 | | 0.1 | 0.8 |
|  | Fe ^2+^ ( mg/L) | bdl | 16.3 | 3.3 | 3.4 | bdl | 0.1 | bdl | bdl | bdl | 0.6 | 0.1 | 0.1 | bdl | bdl | bdl | | bdl | bdl |
| DOC, TN,  Cations, and anions measured in lab^2^ | DOC (mg/L) | 2.0 | 43.9 | 11.1 | 9.8 | 1.7 | 1.9 | 1.5 | 1.6 | 1.7 | 1.9 | 1.7 | 1.9 | 2 | 1.7 | 1.5 | | 1.5 | 1.6 |
|  | Total N (mg/L) | 0.4 | 16.8 | 2.8 | 4.5 | 0.3 | 0.4 | 0.4 | 0.3 | 0.3 | 4.1 | 0.4 | 0.7 | 0.4 | 0.4 | 0.4 | | 0.3 | 0.4 |
|  | Cl^-^ (mg/L) | 825.8 | 8.2 | bdl | 6.0 | 646.6 | 672.9 | 658.3 | 702.6 | 710.3 | 36.1 | 618.1 | 599.7 | 382.4 | 374.2 | 166.3 | | 92.8 | 146.9 |
|  | SO_4_^2-^ (mg/L) | 13.2 | 243.2 | 809.9 | 991.2 | 17.2 | 21.4 | 23.6 | 20.4 | 19.8 | 103.9 | 18.5 | 18.9 | 20.7 | 21.3 | 23.6 | | 2.1 | 22.2 |
|  | Br^-^ (mg/L) | 3.1 | bdl | bdl | bdl | 5.4 | bdl | bdl | 5.4 | 2.8 | bdl | 2.4 | 3.4 | 2.5 | 0.8 | bdl | | bdl | bdl |
|  | F^-^ (mg/L) | 15.9 | bdl | bdl | 0.3 | 12.4 | 13.7 | 13.0 | 14.7 | 14.1 | bdl | 11.7 | 12.1 | 7.2 | 5.4 | 4.8 | | 12.9 | 5.1 |
|  | NO_3_^-^ (mg/L) | 6.8 | bdl | bdl | bdl | bdl | bdl | bdl | bdl | bdl | 1.1 | 0.6 | bdl | bdl | bdl | bdl | | bdl | bdl |
|  | PO_4_^3-^ (mg/L) | bdl | bdl | bdl | 2.6 | 0.3 | bdl | bdl | bdl | bdl | bdl | bdl | bdl | bdl | bdl | bdl | | bdl | bdl |
|  | Mg (mg/L) | 0.1 | 0.2 | 1.2 | 6.7 | bdl | bdl | bdl | 0.1 | 0.1 | 0.3 | 0.1 | 0.1 | 0.2 | 0.1 | 4.2 | | 4.1 | 4.0 |
|  | Si (mg/L) | 52.7 | 9.1 | 19.4 | 16.7 | 98.7 | 104.4 | 76.9 | 89.2 | 94.3 | 27.4 | 66.8 | 52.7 | 60.2 | 67.6 | 46.4 | | 43.7 | 50.0 |
|  | Fe (mg/L) | 0.3 | 4.6 | 26.8 | 16.4 | 0.6 | bdl | bdl | bdl | 0.54 | 0.2 | 1.2 | 1.0 | bdl | 0.1 | bdl | | bdl | bdl |
|  | Ti (mg/L) | bdl | bdl | bdl | bdl | bdl | bdl | bdl | bdl | bdl | bdl | 0.1 | bdl | 0.1 | bdl | bdl | | bdl | 0.1 |
|  | Al (mg/L) | 0.2 | 6.0 | 43.2 | 40.4 | 0.3 | 0.7 | 0.3 | 0.9 | 0.8 | 0.3 | 1 | 0.5 | 0.4 | 0.5 | 0.1 | | bdl | 0.4 |
|  | Ca (mg/L) | bdl | 1.5 | 12.8 | 48.7 | 0.4 | 0.2 | bdl | 0.6 | 0.4 | 2.2 | 1.0 | 0.9 | 1.9 | 2.0 | 6.9 | | 9.2 | 8.2 |
|  | K (mg/L) | 154.2 | 32.8 | 39.8 | 92.7 | 128.7 | 130.4 | 126.8 | 134.2 | 134.6 | 34.2 | 124.5 | 105.7 | 66.8 | 78.2 | 46.1 | | 45.9 | 42.6 |
|  | Na (mg/L) | 862.2 | 5.3 | 11.2 | 17.5 | 717.9 | 721.3 | 719.9 | 738.8 | 752.8 | 46.9 | 701.4 | 628.3 | 414.0 | 475.9 | 430.0 | | 418.5 | 390.2 |
| Sediment/sinter  Geochemi.^3^ | TOC (mg/g)^4^ | 0.5 | 2.6 | 1.7 | 0.9 | 0.8 | 1.0 | 1.2 | 0.6 | 1.4 | 0.9 | bdl | bdl | 0.1 | bdl | 0.7 | | 4.4 | 2.5 |
|  | TN (mg/g) | 0.1 | 0.2 | 0.1 | 0.1 | 0.1 | 0.1 | 0.1 | 0.1 | 0.2 | 0.2 | bdl | bdl | bdl | bdl | bdl | | bdl | bdl |
|  | quartz |  | +++ | +++ | +++ | +++ | +++ | +++ | +++ | +++ | +++ |  |  | ++ | +++ |  | |  | ++ |
|  | calcite |  |  |  |  |  |  |  | + |  |  |  |  |  |  | +++ | | ++ | +++ |
|  | albite |  |  |  |  |  |  | ++ |  |  |  |  |  | ++ | + |  | |  |  |
|  | microcline |  |  |  |  | ++ |  | + | + | ++ |  |  |  | ++ | ++ |  | |  | ++ |
|  | zirconolite |  | + |  |  |  |  |  |  |  |  |  |  |  |  |  | |  |  |
|  | arsenopyrite |  |  |  | + |  |  |  |  |  |  |  |  |  |  |  | |  |  |
|  | clinochlore |  |  |  |  |  | + |  |  |  |  |  |  |  |  |  | |  |  |
|  | goethite |  |  |  |  |  | ++ |  |  |  |  |  |  |  |  |  | |  |  |
|  | aragonite |  |  |  |  |  | ++ |  |  |  |  |  |  |  |  | + | | +++ |  |
|  | forsterite |  |  |  |  |  |  |  |  | + |  |  |  |  |  |  | |  |  |
|  | troilite |  |  |  |  |  |  |  |  |  |  |  |  |  |  |  | | ++ |  |
|  | periclase |  |  |  |  |  |  |  |  |  |  |  |  |  |  |  | | ++ |  |
|  | ewaldite |  |  |  |  |  |  |  |  |  |  |  |  |  |  |  | | + |  |
|  | wuestite |  |  |  |  |  |  |  |  |  |  |  |  |  |  |  | |  | + |
|  | muscovite |  |  |  |  |  |  |  |  |  |  |  |  | + |  |  | |  |  |
|  | witherite |  |  |  |  |  |  |  |  |  |  |  |  | + |  |  | |  |  |

^1^ Dgg: Dagunguo – Great Boiling Spring, Drty-1 - Diretiyanqu - Experimental Site (just under a cliff), Drty- 2 - Diretiyanqu - Experimental Site (down the spring Drty1), Drty-3 - Diretiyanqu - Experimental Site (Right to the spring Drty1), GmqS - Guminquan– Drum Beating Spring (source); GmqC - Guminquan – Drum Beating Spring (right channel); GmqP- Guminquan – Drum BeatingSpring (streamer pool); JmqL - Jimeiquan - Sisters Spring (Left); JmqR - Jimeiquan - Sisters Spring (Right); Zzq - Zhenzhuquan - Pearl Spring; HtjL - Huitaijing - Pregnancy Well (Left);

HtjR - Huitaijing - Pregnancy Well (Right); SrbzU - Shuirebaozhaqu – HydrothermalOutbreak (upstream); SrbzD - Shuirebaozhaqu - Hydrothermal Outbreak (downstream);

GxsS - Gongxiaoshe - Coop Hotel (side); GxsB – Gongxiaoshe - Coop Hotel (bottom); Jz – Jinze – Golden Pond Motel.

^2^ bdl: below detection limit.

^3^ The semi-quantitative abundances of minerals were designated with “+”, “++”, and “+++”, referring to present (<10%), moderate (10-50%), and abundant (>50%), respectively; XRD pattern for the Dagunguo (Dgg) sinter sample does not show any distinct peaks, and it is impossible to identify its mineralogy.

^4^TOC content is mg C per g carbonate-free sediment.
