# Supplementary material for: A Comprehensive Census of Microbial Diversity in Hot Springs of Tengchong, Yunnan Province China Using 16S rRNA Gene Pyrosequencing
Source: PLoS One. 2013 Jan 9;8(1):e53350. doi: 10.1371/journal.pone.0053350 (PMC3541193; doi:10.1371/journal.pone.0053350)
Supplement: Table S2 — Trace metal concentrations of hot spring water samples from Tengchong (values in ppb). (DOCX) [file pone.0053350.s010.docx]

**Table S2** Trace metal concentrations of hot spring water samples from Tengchong (values in ppb)**

|  | Dgg | Drty-1 | Drty-2 | Drty-3 | GmqS | GmqC | GmqP | JmqL | JmqR | Zzq | HtjR | SrbzU | SrbzD | GxsS | GxsB | Jz |
| --- | --- | --- | --- | --- | --- | --- | --- | --- | --- | --- | --- | --- | --- | --- | --- | --- |
| Be | 6.4 | 4 | 3.1 | 1.4 | 4.3 | 4.7 | 4.3 | 4.9 | 3.3 | 1.19 | 2.62 | 5.2 | 4.2 | 4.4 | 2.87 | 4 |
| Mg | 10.02 | 4900 | 1110 | 231.8 | 25 | 4.4 | 20 | 4.6 | 4.9 | 300 | 6.8 | 118 | 62 | 4840 | 3350 | 3330 |
| P | 4 | 2900 | 343 | 5.7 | 11.3 | 9 | 87 | 6.4 | 3.6 | 3.9 | 14 | 12.9 | 12.1 | 12 | 17 | 14.7 |
| Ti | bdl | 10.5 | 6.7 | 1.94 | bdl | bdl | 0.25 | bdl | bdl | 0.17 | bdl | bdl | bdl | bdl | bdl | bdl |
| V | 0.85 | 6.3 | 2.17 | 0.9 | 0.59 | 0.71 | 0.66 | 0.54 | 0.36 | 0.84 | 0.54 | 0.50 | 0.45 | 0.68 | 0.366 | 0.616 |
| Cr | bdl | 14.8 | 0.96 | 0.25 | bdl | bdl | bdl | bdl | bdl | 0.059 | bdl | bdl | bdl | bdl | 0.12 | bdl |
| Mn | 2.1 | 1010 | 249 | 61 | 5.7 | 5.6 | 7.4 | 5.7 | 4.6 | 152 | 7.9 | 24.0 | 13.57 | 14.5 | 10.3 | 22 |
| Fe | 5.1 | 15600 | 24300 | 4340 | 2.6 | 3.2 | 16.4 | 3.16 | 1.48 | 580 | 2.3 | 23.6 | 15.1 | 255 | 175 | 105 |
| Co | bdl | 3.7 | 2.46 | 0.7 | bdl | bdl | bdl | bdl | bdl | 0.137 | bdl | bdl | bdl | bdl | bdl | 0.16 |
| Ni | 0.14 | 9.6 | 3.7 | 21 | 0.15 | 0.3 | 0.9 | 0.1 | 0.1 | 0.34 | 0.1 | 0.222 | 0.19 | 0.15 | 0.28 | 0.3 |
| Cu | 0.1 | 1.5 | 1.73 | 2.23 | 0.09 | 0.09 | 0.45 | 0.07 | bdl | 0.12 | 0.06 | 0.054 | 0.066 | 0.08 | 0.07 | 0.054 |
| Zn | 1.2 | 610 | 88 | 58.6 | 3.2 | 1.8 | 5.7 | 0.6 | 1.05 | 11.7 | 2.3 | 0.75 | 0.83 | 2.1 | 3.71 | 4.7 |
| Ga | 2.3 | 4.6 | 2.23 | 0.77 | 2.5 | 2.41 | 2.5 | 2.8 | 1.87 | bdl | 2.4 | 0.51 | 0.57 | 0.41 | 0.3 | 0.15 |
| Ge | 43 | 2.4 | 2.2 | 1.8 | 36.9 | 34 | 35 | 36 | 28 | 2.4 | 35 | 20 | 21 | 18 | 11.6 | 10.8 |
| As | 430 | 122 | 190 | 59 | 500 | 470 | 470 | 470 | 400 | 51 | 430 | 160 | 200 | 980 | 580 | 500 |
| Se | 0.9 | bdl | bdl | 0.2 | bdl | bdl | bdl | bdl | bdl | bdl | bdl | bdl | bdl | bdl | bdl | bdl |
| Rb | 1430 | 660 | 445 | 350 | 1210 | 1380 | 1210 | 1270 | 920 | 240 | 1230 | 770 | 690 | 460 | 310 | 303 |
| Sr | 79.6 | 36 | 10.6 | 5.58 | 66 | 66 | 68.1 | 79 | 56 | 5.7 | 42.7 | 33.5 | 24.3 | 810 | 550 | 581 |
| Y | 0.015 | 28 | 39.3 | 6.7 | 0.016 | 0.02 | 0.056 | 0.016 | 0.02 | 0.67 | 0.068 | 0.075 | 0.051 | 0.027 | 0.024 | 0.03 |
| Zr | 0.03 | 0.226 | 0.102 | 0.064 | 0.044 | 0.044 | 0.08 | 0.032 | 0.036 | 0.15 | 0.08 | 0.075 | 0.105 | 0.054 | 0.042 | 0.39 |
| Nb | 0.025 | 0.292 | 0.116 | 0.028 | 0.02 | 0.028 | 0.024 | 0.024 | 0.02 | bdl | 0.024 | 0.018 | 0.015 | bdl | bdl | bdl |
| Mo | 0.12 | 0.218 | 0.104 | 0.14 | 0.11 | 0.1 | 0.13 | 0.084 | 0.072 | 0.029 | 0.116 | 0.06 | 0.048 | 0.24 | 0.153 | 0.166 |
| Ru | bdl | 0.006 | 0.006 | 0.004 | bdl | bdl | bdl | bdl | bdl | bdl | 0.008 | 0.006 | 0.006 | 0.009 | bdl | 0.006 |
| Rh | 0.005 | 0.004 | 0.004 | 0.002 | 0.004 | 0.004 | 0.004 | 0.004 | 0.004 | 0.001 | 0.008 | 0.003 | 0.006 | 0.015 | 0.009 | 0.01 |
| Pd | 0.055 | 0.66 | 0.85 | 0.17 | 0.04 | 0.04 | 0.04 | 0.044 | 0.032 | 0.018 | 0.04 | 0.03 | 0.033 | 0.135 | 0.096 | 0.088 |
| Ag | 0.03 | 0.012 | 0.01 | 0.01 | 0.04 | 0.032 | 0.108 | 0.028 | bdl | bdl | 0.02 | bdl | bdl | 0.018 | bdl | bdl |
| Cd | bdl | 0.38 | 0.18 | 0.078 | bdl | bdl | bdl | bdl | bdl | 0.012 | bdl | bdl | bdl | bdl | bdl | bdl |
| Sn | bdl | 0.388 | 0.054 | 0.038 | 0.024 | 0.028 | 0.064 | bdl | bdl | 0.011 | 0.036 | bdl | bdl | 0.015 | bdl | bdl |
| Sb | 26.7 | 1.78 | 5.4 | 1.72 | 23.1 | 24.3 | 22.5 | 22.8 | 16.3 | 0.55 | 20 | 8.22 | 8 | 10.7 | 7.1 | 7.23 |
| Cs | 770 | 45.7 | 48 | 29 | 630 | 680 | 640 | 690 | 486 | 33 | 610 | 396 | 367 | 750 | 490 | 486 |
| Ba | 2.05 | 33.9 | 9.7 | 8.6 | 2.9 | 3.03 | 3.15 | 1.9 | 1.28 | 6.1 | 3 | 42.6 | 36.3 | 790 | 510 | 640 |
| La | bdl | 7.4 | 4.9 | 4.3 | bdl | bdl | 0.028 | bdl | bdl | 0.31 | bdl | 0.021 | 0.015 | 0.015 | 0.012 | 0.01 |
| Ce | bdl | 20.4 | 16.5 | 11.7 | bdl | 0.012 | 0.068 | bdl | 0.016 | 0.75 | 0.028 | 0.048 | 0.033 | bdl | 0.009 | 0.008 |
| Pr | bdl | 2.88 | 2.77 | 1.51 | bdl | bdl | bdl | bdl | bdl | 0.106 | bdl | 0.009 | bdl | bdl | bdl | bdl |
| Nd | bdl | 13.4 | 13.6 | 6.3 | bdl | bdl | 0.04 | bdl | bdl | 0.48 | bdl | 0.039 | 0.021 | bdl | bdl | bdl |
| Sm | bdl | 4.58 | 5.2 | 1.74 | bdl | bdl | bdl | bdl | bdl | 0.132 | bdl | bdl | bdl | bdl | bdl | bdl |
| Eu | bdl | 0.326 | 0.33 | 0.102 | bdl | bdl | bdl | bdl | bdl | 0.009 | bdl | bdl | bdl | 0.072 | 0.045 | 0.056 |
| Gd | bdl | 4.53 | 5.32 | 1.47 | bdl | bdl | bdl | bdl | bdl | 0.107 | bdl | bdl | bdl | bdl | bdl | bdl |
| Tb | bdl | 0.87 | 1.074 | 0.246 | bdl | bdl | bdl | bdl | bdl | 0.017 | bdl | bdl | bdl | bdl | bdl | bdl |
| Dy | bdl | 5.4 | 6.76 | 1.42 | bdl | bdl | bdl | bdl | bdl | 0.098 | bdl | bdl | bdl | bdl | bdl | bdl |
| Ho | bdl | 1.07 | 1.372 | 0.284 | bdl | bdl | bdl | bdl | bdl | 0.021 | bdl | bdl | bdl | bdl | bdl | bdl |
| Er | bdl | 3.32 | 4.26 | 0.91 | bdl | bdl | bdl | bdl | bdl | 0.073 | bdl | 0.009 | bdl | bdl | bdl | bdl |
| Tm | bdl | 0.518 | 0.676 | 0.142 | bdl | bdl | bdl | bdl | bdl | 0.013 | bdl | bdl | bdl | bdl | bdl | bdl |
| Yb | bdl | 3.56 | 4.59 | 0.97 | bdl | bdl | bdl | bdl | bdl | 0.1 | bdl | bdl | bdl | bdl | bdl | bdl |
| Hf | bdl | 0.01 | 0.01 | 0.004 | bdl | bdl | bdl | bdl | bdl | bdl | bdl | bdl | bdl | bdl | bdl | 0.01 |
| Ta | bdl | 0.006 | 0.004 | 0.002 | bdl | bdl | bdl | bdl | bdl | bdl | bdl | bdl | bdl | bdl | bdl | bdl 4 |
| W | 66 | 0.164 | 0.37 | 0.076 | 52 | 57 | 52.8 | 57 | 40.3 | 1.22 | 51 | 32 | 29.3 | 86 | 56 | 52.9 |
| Re | bdl | bdl | bdl | bdl | bdl | bdl | bdl | bdl | bdl | bdl | bdl | bdl | bdl | bdl | bdl | bdl |
| Os | bdl | 0.004 | 0.002 | 0.006 | bdl | bdl | bdl | bdl | bdl | bdl | bdl | bdl | bdl | bdl | bdl | bdl |
| Pt | 0.005 | 0.002 | 0.002 | 0.002 | 0.004 | 0.004 | 0.004 | 0.004 | 0.004 | 0.001 | 0.004 | 0.003 | 0.003 | 0.003 | 0 | 0.002 |
| Au | 0.29 | 0.044 | 0.032 | 0.02 | 0.19 | 0.196 | 0.22 | 0.176 | 0.164 | 0.031 | 0.18 | 0.162 | 0.144 | 0.078 | 0.066 | 0.1 |
| Tl | 6.4 | 5.39 | 5.11 | 3.08 | 5.3 | 5.6 | 5.2 | 5.4 | 4 | 0.79 | 4.8 | 1.89 | 1.9 | 1.37 | 0.94 | 1.14 |
| Pb | 0.02 | 8.29 | 10.8 | 5 | 0.056 | 0.06 | 0.184 | 0.032 | 0.028 | 0.25 | 0.052 | 0.09 | 0.075 | 0.072 | 0.069 | 0.098 |
| Bi | bdl | 0.034 | 0.058 | 0.06 | bdl | bdl | bdl | bdl | bdl | bdl | bdl | bdl | bdl | bdl | bdl | bdl |
| Th | bdl | 11.9 | 23.7 | 3.6 | 0.02 | 0.024 | 0.048 | 0.016 | 0.02 | 0.033 | 0.036 | 0.075 | 0.108 | 0.036 | 0.036 | 0.33 |
| U | bdl | 11.8 | 35.8 | 2.65 | bdl | 0.032 | 0.168 | 0.088 | 0.092 | 4 | 0.076 | 0.429 | 0.297 | bdl | bdl | 0.214 |

**note: bdl means below detection limit; the trace metal contents of spring water at Huitaijing – left (HtjL) was not determined.

The sample codes as in Table S1.
