# Supplementary material for: A Comprehensive Census of Microbial Diversity in Hot Springs of Tengchong, Yunnan Province China Using 16S rRNA Gene Pyrosequencing
Source: PLoS One. 2013 Jan 9;8(1):e53350. doi: 10.1371/journal.pone.0053350 (PMC3541193; doi:10.1371/journal.pone.0053350)
Supplement: Table S3 — Significance tests of the overall microbial community structure between the two primer sets with three different statistical approaches. (DOCX) [file pone.0053350.s011.docx]

**Table S3** Significance tests of the overall microbial community structure between the two primer sets with three different statistical approaches

|  | Adonis ^b^ | | ANOSIM ^c^ | | MRPP ^d^ | |
| --- | --- | --- | --- | --- | --- | --- |
| Data sets^a^ | F | P-value | R | P-value | δ | P-value |
| Original primer vs. Modified primer | 0.23546 | 0.965 | -0.138 | 0.97 | 0.1553 | 0.979 |

Abbreviations: ANOSIM, analysis of similarity; MRPP, multi response permutation procedure.

^a^ Unweighted Unifrac matrix based on the community structure were used for all three non-parametric multivariate analyses.

^b^ Permutational multivariate analysis of variance using distance matrices. Significance tests were performed by F-tests based on sequential sums of squares from permutations of the raw data.

^c^ ANOSIM: Analysis of similarities (ANOSIM) provides a way to test statistically whether there is a significant difference between two or more groups of sampling units. Statistic R is based on the difference of mean ranks between groups and within groups (R ranges from -1 to +1, close to 0 means no differences among groups; R close to ±1 means groups differ in community composition). The significance of observed R is assessed by permuting the grouping vector to obtain the empirical distribution of R under the null model.

^d^ MRPP: Similar to ANOSIM. Statistic delta is the weighted average of within-group distances (Geometrically, a smaller value of the MRPP statistic indicates higher within-group concentration and larger between-group differences). The significance test is the fraction of permuted deltas that are less than the observed delta.
