# Supplementary material for: A Comprehensive Census of Microbial Diversity in Hot Springs of Tengchong, Yunnan Province China Using 16S rRNA Gene Pyrosequencing
Source: PLoS One. 2013 Jan 9;8(1):e53350. doi: 10.1371/journal.pone.0053350 (PMC3541193; doi:10.1371/journal.pone.0053350)
Supplement: Table S4 — Comparison of microbial communities retrieved with the two primer sets. (DOCX) [file pone.0053350.s012.docx]

**Table S4** Comparison of microbial communities retrieved with the two primer sets.

|  | Taxa Levels | | No of Genera | | No of Families | | No of Orders | | No of Classes | | No of Phyla | |
| --- | --- | --- | --- | --- | --- | --- | --- | --- | --- | --- | --- | --- |
| Sample ID | Arch. Percen. (%) | | Arch. | Bact. | Arch. | Bact. | Arch. | Bact. | Arch. | Bact. | Arch. | Bact. |
| Dgg.TFF | Prim^1^ | 0.15 | 3 | 3 | 2 | 2 | 2 | 2 | 2 | 2 | 1 | 2 |
|  | Prim.P | 0. 02 | 2 | 5 | 2 | 4 | 1 | 4 | 1 | 4 | 1 | 3 |
| Drty-3.Sed | Prim | 15.95 | 10 | 13 | 9 | 12 | 7 | 11 | 3 | 11 | 2 | 7 |
|  | Prim.P | 9.36 | 6 | 12 | 5 | 11 | 3 | 11 | 2 | 11 | 2 | 7 |
| GmqP.Str | Prim | 3.31 | 7 | 11 | 5 | 10 | 4 | 10 | 2 | 9 | 2 | 8 |
|  | Prim.P | 0.26 | 3 | 6 | 2 | 5 | 2 | 5 | 1 | 5 | 1 | 4 |
| JmqL.Wat | Prim | 16.91 | 5 | 52 | 4 | 46 | 3 | 40 | 2 | 29 | 1 | 17 |
|  | Prim.P | 20.04 | 6 | 23 | 5 | 20 | 3 | 19 | 1 | 18 | 1 | 13 |
| SrbzD.Sed | Prim | 18.27 | 12 | 44 | 7 | 39 | 4 | 35 | 4 | 24 | 2 | 17 |
|  | Prim.P | 7.75 | 8 | 22 | 7 | 21 | 5 | 18 | 3 | 16 | 2 | 14 |
| GxsS.Wat | Prim | 0.04 | 2 | 6 | 2 | 6 | 2 | 6 | 1 | 6 | 1 | 6 |
|  | Prim.P | 0.20 | 2 | 8 | 2 | 8 | 2 | 8 | 1 | 8 | 1 | 8 |
| GxsB.Sin2 | Prim | 11.22 | 4 | 37 | 4 | 33 | 4 | 33 | 3 | 30 | 1 | 22 |
|  | Prim.P | 2.78 | 2 | 41 | 2 | 38 | 2 | 37 | 2 | 32 | 1 | 23 |

^1^The label “Prim” represents the modified primer set, and the label “Prim.P” represents the unmodified primer set.
