# Supplementary material for: A Comprehensive Census of Microbial Diversity in Hot Springs of Tengchong, Yunnan Province China Using 16S rRNA Gene Pyrosequencing
Source: PLoS One. 2013 Jan 9;8(1):e53350. doi: 10.1371/journal.pone.0053350 (PMC3541193; doi:10.1371/journal.pone.0053350)
Supplement: Supplementary file 6 [file pone.0053350.s003.docx]

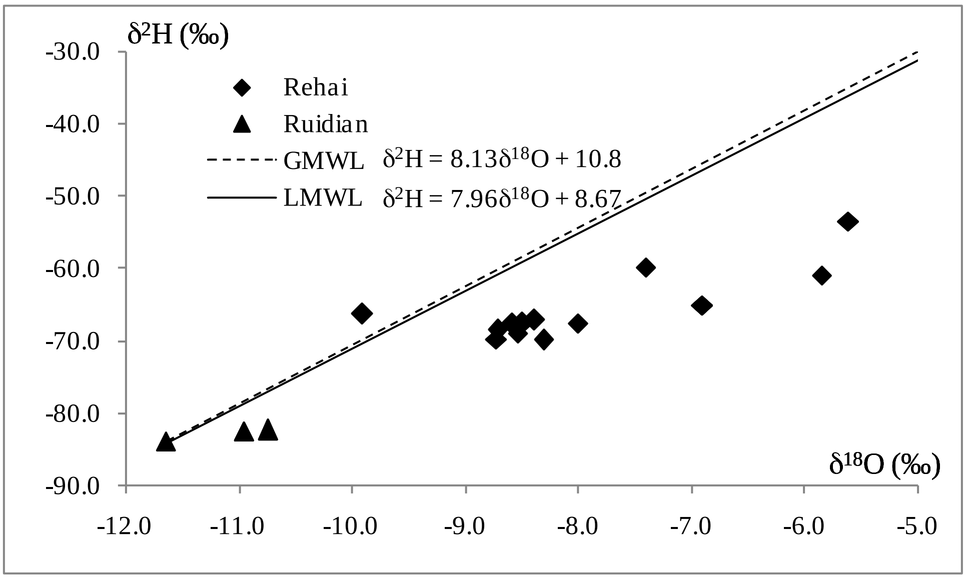
δ²H (‰)
Rehai
Ruidian
GMWL δ²H = 8.13δ¹⁸O + 10.8
LMWL δ²H = 7.96δ¹⁸O + 8.67
δ¹⁸O (‰)
-30.0
-40.0
-50.0
-60.0
-70.0
-80.0
-90.0
-12.0
-11.0
-10.0
-9.0
-8.0
-7.0
-6.0
-5.0
